# A Systematic Evaluation of Multi-Gene Predictors for the Pathological Response of Breast Cancer Patients to Chemotherapy

**DOI:** 10.1371/journal.pone.0049529

**Published:** 2012-11-21

**Authors:** Kui Shen, Nan Song, Youngchul Kim, Chunqiao Tian, Shara D. Rice, Michael J. Gabrin, W. Fraser Symmans, Lajos Pusztai, Jae K. Lee

**Affiliations:** 1 Precision Therapeutics, Inc., Pittsburgh, Pennsylvania, United States of America; 2 Department of Public Health Sciences, University of Virginia, Charlottesville, Virginia, United States of America; 3 Department of Pathology, University of Texas MD Anderson Cancer Center, Houston, Texas, United States of America; 4 Division of Breast Medical Oncology, Yale Cancer Center, New Haven, Connecticut, United States of America; University of Texas MD Anderson Cancer Center, United States of America

## Abstract

Previous studies have reported conflicting assessments of the ability of cell line-derived multi-gene predictors (MGPs) to forecast patient clinical outcomes in cancer patients, thereby warranting an investigation into their suitability for this task. Here, 42 breast cancer cell lines were evaluated by chemoresponse tests after treatment with either TFAC or FEC, two widely used standard combination chemotherapies for breast cancer. We used two different training cell line sets and two independent prediction methods, superPC and COXEN, to develop cell line-based MGPs, which were then validated in five patient cohorts treated with these chemotherapies. This evaluation yielded high prediction performances by these MGPs, regardless of the training set, chemotherapy, or prediction method. The MGPs were also able to predict patient clinical outcomes for the subgroup of estrogen receptor (ER)-negative patients, which has proven difficult in the past. These results demonstrated a potential of using an *in vitro*-based chemoresponse data as a model system in creating MGPs for stratifying patients’ therapeutic responses. Clinical utility and applications of these MGPs will need to be carefully examined with relevant clinical outcome measurements and constraints in practical use.

## Introduction

Breast cancer remains a significant cause of mortality in women with nearly 40,000 deaths in the U.S. during 2010 alone [1]. Although neoadjuvant chemotherapy is widely used in the treatment of early-stage breast cancer [2], [3], [4], selecting the best treatment regimen for an individual patient from several options is not straightforward, as the response to chemotherapy varies considerably among patients–even those with cancers exhibiting identical histological and molecular subtypes [5], [6], [7]. Gene expression profiling studies have provided a molecular classification of breast cancer into clinically relevant subtypes and new tools to predict disease recurrence and response to different treatments. Recently, efforts have been undertaken to develop multi-gene predictors (MGPs) of drug response using a patient’s own tissue samples in the hopes that the predictors might guide treatment decisions [8], [9], [10], [11]. In order to create such MGPs, large numbers of clinically homogenous patient samples are required, the acquisition of which is a costly, lengthy, and invasive process. Moreover, creating MGPs using samples from patients undergoing the same treatment limits their utility when predictors for multiple standard-of-care treatments are necessary.

Recently, researchers have attempted to overcome the limitations of patient sample-derived MGPs by using cancer cell lines to develop MGPs, with varying degrees of success [12], [13], [14], [15], [16], [17], [18]. A recent study of 51 breast cancer cell lines by Neve, et al. reported that these cell lines mirror many, though not all, of the biological and genomic properties of primary tumors, which suggests the possibility of utilizing cell line-derived MGPs as surrogates for homogeneous patient samples [18]. Shen et al. also demonstrated the prediction capability of breast cancer cell line-derived MGPs, which were validated in blinded clinical trials (US Oncology 02-103 and NSABP B-27), suggesting the feasibility of using breast cancer cell lines to develop genomic predictors of response to neoadjuvant chemotherapy [19], [20]. In addition, Kadra, et al. recently determined gene signatures in response to taxanes and ixabepilone in breast cancer cell lines and achieved promising performance when the resulting predictors were applied to publicly available clinical trial data [21]. However, other studies have not reported such success. For example, when Baggerly, et al. examined five case studies, they found that approaches using the *in vitro* drug response of NCI60 cell lines to predict patient chemotherapy response were not successful [22]. Liedtke, et al. used 19 breast cancer cell lines to create MGPs for four commonly used chemotherapies, but these did not accurately predict patient responses [15].

These conflicting data on the utility of cell line-derived MGPs highlights the need for further and complete evaluation, including for those MGPs developed from breast cancer cell lines. Many factors, including the precision of the *in vitro* assay, the selection and number of cell lines, the platform and quality of array measurements, and the statistical method employed, may contribute to this discrepancy. To address these questions, two different sets of breast cancer cell lines were exposed to two combination chemotherapies–TFAC (paclitaxel, 5-fluorouracil, doxorubicin, and cyclophosphamide) and FEC (5-fluorouracil, epirubicin, and cyclophosphamide)–and assayed by an *in vitro* chemoresponse test. We also independently developed our MGPs using two prediction methods, supervised principal component regression (superPC) and CO-eXpression ExtrapolatioN (COXEN), developed by the groups at Precision Therapeutics, Inc. and the University of Virginia, respectively. We subsequently validated these MGPs in five clinical trials with patient gene expression profiling data and full clinical annotation of chemotherapy treatment and outcome. The goal of this systematic investigation was to objectively evaluate the effectiveness of cell line-derived MGPs as tools to guide clinical decisions in the application of standard chemotherapies.

## Materials and Methods

### A Chemoresponse Test for Breast Cancer Cell Lines

Forty-two breast cancer cell lines (**Table S1**) were obtained from either ATCC (Manassas, VA) or DSMZ (Braunschweig, Germany). RPMI 1640 medium (Mediatech, Herndon, VA) containing 10% FBS (HyClone, Logan, UT) was used to maintain all of the cell lines at 37°C in 5% CO_2_. Before conducting *in vitro* chemoresponse tests, each cell line was trypsinized and seeded into 384-well microtiter plates (Corning, Lowell, MA) after reaching roughly 80% confluence.

Ten serial dilutions, in triplicate, of the TFAC combination of paclitaxel (T, 0.2–100 nM), 5-fluorouracil (F, 0.1–50 µM), doxorubicin (A, 2 nM–1.2 µM), and pre-activated cyclophosphamide (4-hydroperoxycyclophosphamide, C, 0.2–13.6 µM), or the FEC combination of 5-fluorouracil (F, 0.1–50 µM), epirubicin (E, 0.7 nM–13.5 µM), and pre-activated cyclophosphamide (4-hydroperoxycyclophosphamide, C, 0.2 µM–13.6 µM) plus media controls were prepared in 10% RPMI 1640 medium before being used to treat each cell line. Both combination treatments were composed of equal volumes of each drug at each dose. The cells were incubated at 37°C in 5% CO_2_ for 72 hours. The chemoresponse test was performed as previously described [23]. Briefly, non-adherent cells and medium were first removed from each well, and the remaining adherent cells were fixed in 95% ethanol and stained with DAPI (Molecular Probes, Eugene, OR). The number of stained cells remaining after drug treatment was determined by a proprietary automated microscope [24], and the survival fraction (SF) calculated as
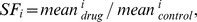
where 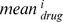 is the average of the number of surviving cells in the drug-treated wells at dose *i*, and 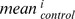 is the average number of living cells in the control wells at dose *i*. The area under the dose-response curve (AUD), 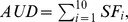 was calculated to quantify the sensitivity of each cell line to the TFAC or FEC treatment. A lower AUD score generated by cell lines represents a greater chemosensitivity to TFAC or FEC.

### Breast Cancer Cell Line Data

The gene expression omnibus database (http://www.ncbi.nlm.nih.gov/geo/, accession number GSE12777, labeled here as “Hoeflich” [17]) and the European Bioinformatics Institute database (http://www.ebi.ac.uk/, accession number E-TABM-157, labeled here as “Neve” [18]) were accessed to obtain gene expression profiles for the 42 breast cancer cell lines generated by the Affymetrix HG-U133 Plus 2.0 Array (Affymetrix, Santa Clara, CA, **Table 1**). Probe-level intensities were generated by the RMAExpress V1.05 software package (http://rmaexpress.bmbolstad.com/) using default settings, except that the probe-level model analysis method was used to summarize probe values. Before further analyses, the probe-level intensities were log_2_-transformed. Probe sets that had low levels of variation (interquartile range <0.5) or low expression values (median<log_2_ [100]) were non-specifically filtered out across all cell lines. The expression values were then standardized to a mean of zero and a standard deviation of one for each cell line.

**Table 1:** Two breast cancer cell line sets for MGP model training.

Database source	GEO/EBI accession number	Microarray platform	number of breast cancer cell lines	
			TFAC	FEC
Hoeflich [17]	GSE12777	HG-U133 Plus 2.0	41^a^	39^b^
Neve [18]	E-TABM-157	HG-U133A	30^c^	30
Common cell lines between Neve and Hoeflich data sets			28^d^	27

a The TFAC chemoresponse test for one cell line (SW527) did not pass quality control; therefore, the AUC values for 41 cell lines were available for further analysis.

b The FEC chemoresponse test for three cell lines (HCC1419, HCC1569, and HCC1806) did not pass quality control; therefore, the AUC values for 39 cell lines were available for further analysis.

c The number of cell lines common to the Hoeflich data set and the 42 cell lines and whose chemoresponses were measured is 30.

d There are 28 TFAC-treated and 27 FEC-treated cell lines common to the Neve and Hoeflich data sets.

### Breast Cancer Patient Data

To objectively evaluate the performance of our MGPs, gene expression data and clinical outcomes for TFAC and FEC treatments from five independent breast cancer clinical trials were used as test sets (**Table 2**). All patients in these five cohorts received neoadjuvant chemotherapy. The first two cohorts are part of the MicroArray Quality Control (MAQC) breast cancer dataset, who received 6 months of TFAC neoadjuvant chemotherapy. Since the first 130 patients were used as the training dataset and the following 100 patients were used for validation in the original study, these two datasets are referred to as MAQC-training and MAQC-test, respectively. For both of these datasets, there are ∼60% ER+ patients, and ∼40% ER– patients. Patients in the third (Tabchy-TFAC n = 91) and fourth (Tabchy-FEC n = 87) cohorts were accrued by MD Anderson and randomly assigned to receive either weekly paclitaxel×12 cycles followed by FAC×4 or FAC/FEC×6 neoadjuvant chemotherapy. For both datasets, there were ∼55% ER+ patients, and ∼45% ER– patients. The fifth cohort (Iwamoto) included 82 patients, with 50% ER+ and 50% ER–. All patients were treated with four courses of FAC or FEC chemotherapy. For all of these data sets, the gene expression profiles of patients were measured from fine-needle aspiration specimens before chemotherapy treatment. The patient’s pathologic complete response (pCR) was tested after treatment to demonstrate the chemotherapy efficacy.

**Table 2:** Summary information for the gene expression and clinical outcome test sets for five clinical trials in the GEO database.

	MAQC-training	MAQC-validation	Tabchy-TFAC	Tabchy-FEC	Iwamoto
**GEO accession #**	GSE20194	GSE20194	GSE20271	GSE20271	GSE22093^a^
**Neoadjuvant treatment**	TFAC	TFAC	TFAC	FAC/FEC	FAC/FEC
**# of all patients**	130	100	91	87	82
**# of ER+ patients**	80	61	49	49	41
**# of ER− patients**	50	39	41	37	41
**# of pCR patients**	33	15	19	7	24
**# of RD patients**	97	85	72	80	58
**pCR%**	34.00%	17.60%	26.40%	8.80%	42.40%

a The Tabchy-TFAC data set (GSE20271) has 31 samples that overlap with the Iwamoto data set (GSE22093); therefore, these two data sets are not completely independent.

### Development of TFAC and FEC MGPs Using the SuperPC Method

Supervised principal components (superPC) regression was used to develop the MGPs for TFAC and FEC chemotherapies [25]. The resulting MGPs were then implemented using the superPC V1.05 software package (http://www-stat.stanford.edu/~tibs/superpc) under the programming environment R 2.11.1(http://www.r-project.org/). In short, the association between the cell line chemoresponse-derived AUD scores and the expression values for each probe set was analyzed by univariate linear regression analysis. In a linear regression model, the first principal component was chosen to predict the result of patient chemotherapy, as measured by pCR. A lower prediction score corresponded with greater chemotherapy sensitivity and therefore a higher likelihood of achieving pCR. To investigate the impact of the number of predictor genes, this parameter was varied from 50 to 1000.

### Development and Validation of TFAC and FEC MGPs Using the COXEN Method

The COXEN method was also used to develop MGPs, as previously described [13]. In brief, *in* *vitro* chemoresponse data was used for TFAC and FEC combination treatments of the 42 breast cancer cell lines to obtain initial candidate expression biomarkers (probe sets) that were the most predictive of the cell lines’ chemosensitivities to each drug combination. Specifically, initial candidate biomarkers differentially expressed between chemo-sensitive and –resistant cell lines were identified from the breast cancer cell lines by two-sample t-test. In addition, biomarkers highly associated with *in vitro* chemosensitivity were identified by evaluating correlation coefficients between drug sensitivity of each cell lines and gene expression data, both with a false-discovery rate (FDR) <0.05. These chemosensitivity biomarkers were then triaged based on the COXEN coefficient which represents the degree of concordance of expression regulation between the breast cancer cell lines and a cohort of breast cancer patients. The mathematical derivation of COXEN coefficient is based on the so called “correlation of correlations”, which first calculates the expression correlations within each set on the same set of genes of interest for both sets and then evaluates gene-by-gene correlation between the two correlation matrices of the two sets. For the derivation of COXEN coefficients in this study, a gene expression dataset compiled from 251 breast cancer patients [26] was used, which was not used in any other manner for our model development or validation.

Using the final COXEN biomarkers for each drug combination, MGPs were developed by applying within-gene standardization and a cross-validated principal component regression analysis to each cell line training set to avoid any potential statistical over-fitting in large screening molecular-based prediction modeling. The biomarkers were then sequentially involved in regression model training by the order of strength of chemosensitivity association. Several competing, high-performance models with different numbers of candidate biomarkers were obtained from the training sets. These competing models were then evaluated and compared by utilizing two of the five clinical trials (**Table 2**). For this analysis, the MAQC-training and Tabchy-FEC datasets were used for selecting the optimal prediction models for TFAC and FEC, respectively. The performance of prediction models were evaluated by testing the difference of prediction scores between response and non-response patient groups using a non-parametric Wilcoxon rank-sum test. The optimal prediction models determined from the evaluation of the two patient sets were then applied in a prospective manner to the completely independent patient cohorts, MAQC-validation and Tabchy-TFAC datasets for TFAC and Tabchy-FEC and Iwamoto datasets for FEC, in order to objectively assess their prediction performance. Predicted scores were then converted into rank-based percentile scores between zero and one within each training or test set for practical use and interpretation of MGP scores as the relative chemoresponse of each cell line or patient in the population. Thus, a higher predicted score implies a higher percentage of responders in a given population to each drug combination used.

For the development of estrogen receptor (ER)-specific prediction models, we divided the cell lines into ER+ and ER– groups based on ESR1 mRNA expression levels, as described elsewhere [27]. The above analyses of biomarker discovery and prediction modeling were then repeated separately for ER+ and ER– patient subsets using the COXEN method.

## Results

### Chemoresponse Test for Breast Cancer Cell Lines

**Figure 1:**
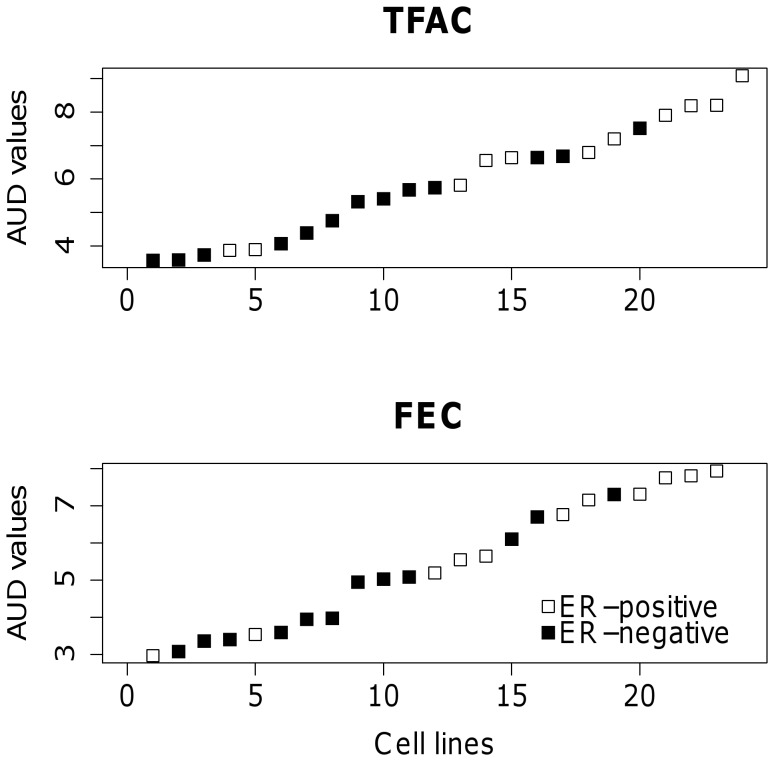
Chemoresponse-derived AUD values for each cell line, labeled by ER status, for both TFAC (top) and FEC (bottom) treatments.

The chemoresponse of each cell line to TFAC and FEC, represented by AUD scores, can be seen graphically in **Figure 1**. For TFAC, the AUD scores ranged from 3.36 to 9.09. For FEC, the AUD scores ranged from 2.81 to 8.00. Generally speaking, the ER+ cell lines demonstrated higher AUD scores (lower chemosensitivity) than the ER– cell lines.

### MGPs Based on Breast Cancer Cell Lines

Using the superPC method, we have developed MGPs for both TFAC and FEC treatments (**Tables S2, S3, S4, S5**). For MGPs developed from both the Neve and Hoeflich training sets, the performance is stable for numbers of genes, in the range 100–700. In this work, we selected the top 150 genes whose expression values are most correlated with the *in vitro* chemosensitivity as gene signatures to predict patient chemotherapy response.

For MGP-FEC and MGP-TFAC, there is substantial overlap no matter which training dataset is used. With Neve used as the training set, the overlap for MGP-TFAC and MGP-FEC was 107 biomarkers, while with Hoeflich used as the training set, the overlap was 80 biomarkers. This was an inevitable result, since TFAC and FEC are very similar chemotherapeutic regimens with two drugs in common (F and C), as well as two drugs that are of the same mechanism of action (A and E). However, the MGPs developed by the Neve and Hoeflich training sets have a relatively small overlap of their biomarkers This may be because these two training sets were measured at different sites and using different platforms (HG-U133 vs HG-U133 plus 2.0). To assess the reproducibility of array data, we calculated the correlation of gene expression based on the 28 cell lines which are common to both training sets, and obtained a correlation of 0.77 for the set of genes that are common in HG-U133 and HG-U133 plus 2.0. The superPC method selects genes based on the association of each gene with drug response; therefore even slight changes in training dataset may lead to a complete different set of genes selected for prediction. Nevertheless, functional analysis indicates that most of these genes are from the same gene functional networks, including cell death, the cell cycle, cellular development, small molecule biochemistry, molecular transport, cellular growth and proliferation, cellular assembly and organization, and cellular function and maintenance. Moreover the scores of predictors generated from Neve and Hoeflich datasets are consistent for all five validation datasets, with the correlation of predictors generated from two training sets all greater than 0.86.

Using the COXEN method, we have also developed MGPs for both TFAC and FEC (**Tables S6, S7, S8, S9**). For the MGPs based on the Neve training set, 47 and 162 COXEN biomarkers were identified for TFAC and FEC, respectively, of which 14 biomarkers were in common. For the MGPs based on the Hoeflich training set, 124 and 20 biomarkers were selected for each TFAC and FEC, and 17 of 20 biomarkers for FEC were also included in those for TFAC. The gene functions of TFAC COXEN biomarkers were primarily from the cell cycle, cellular growth and proliferation, hematological system development, cellular compromise, and drug metabolism and function. Of these, six genes (ABAT, DEPDC1, KIF2C, SMAD4A, TAF1D, and TUBB6) have been reported to be directly relevant to cancer mechanisms, such as mammary tumor progression (P = 0.046) [28], [29], [30], [31]. A majority of FEC COXEN biomarkers were involved in cell death, cancer cellular mechanism, cell morphology, molecular transport, hematological systems and development, as well as DNA replication, recombination, and repair. In particular, 12 genes, including AXL, CDH1, CFLAR, FKBP1A, NT5E, and VIM, were reported to be significantly associated with tumor metastasis (P<8E-04) [28], [29], [30], [31]. Also, 7 genes (CTGF, ESR1, MAPK3, PIK3R3, PLAU, PRNP, and RET) were reported to be highly relevant to growth (P<1E-04) and microtubule dynamics (P<4E-05) in tumor cell lines, and apoptosis in breast cancers (P<1E-04) [32], [33], [34], [35].

### Evaluation of MGPs Developed by All Breast Cancer Cell Lines

**Figure 2:**
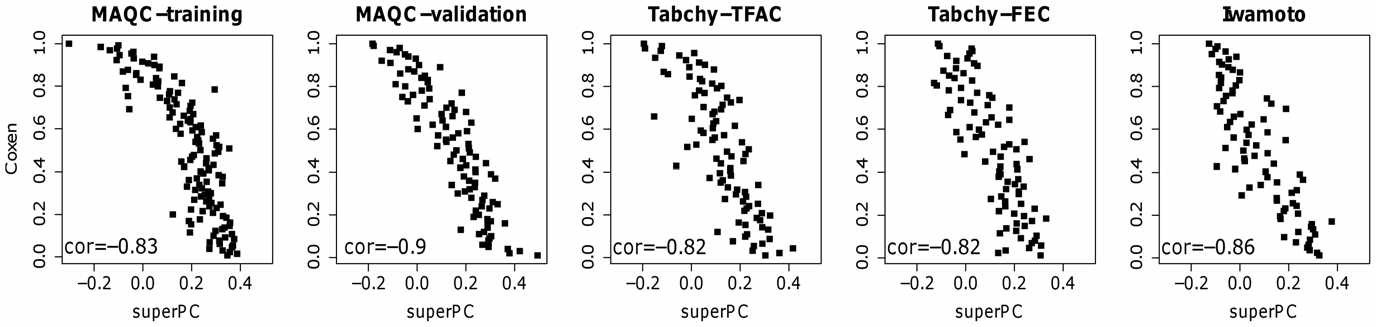
Correlation between the prediction scores calculated by the superPC and COXEN methods from the Neve training set for each test set.

**Figure 3:**
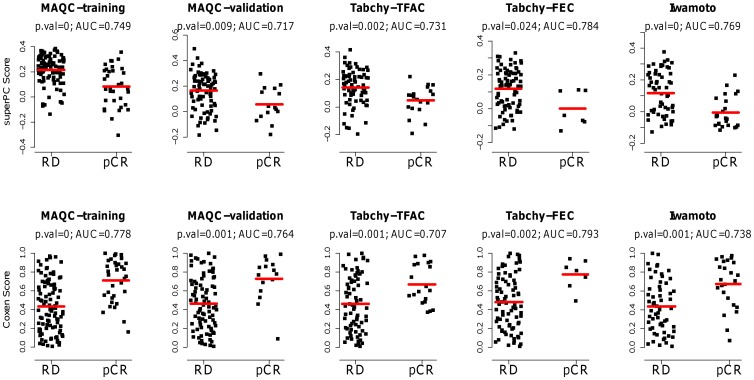
Distributional difference between prediction scores calculated by the superPC or COXEN methods from the Neve training set for responders (pCR, pathologic complete response) and non-responders (RD, residual disease), with p-value and AUC. In the top row, the prediction scores are calculated by the superPC method, and in the bottom row, the prediction scores are calculated by the COXEN method. Red lines represent median prediction scores in each group.

The performance of MGPs was validated by data from five clinical trials, comprised of microarray data from tumors biopsied before the initiation of therapy for each breast cancer patient in the cohort. The prediction scores generated by the MGPs are reported for each clinical trial test set for the superPC and COXEN methods and for both TFAC and FEC treatments. We found that superPC and COXEN prediction scores were highly consistent for both Neve (top row) and Hoeflich (bottom row) training sets. The two prediction methods provided highly correlated scores for both TFAC and FEC chemotherapies with Spearman correlation coefficients between 0.75 and 0.91 (p-value <10^−6^) for all cases, which included different training sets, treatments, and test sets (**Figure 2**). Moreover, predictors developed from the Neve training set distribute significantly different in responders vs non-responders for all five trials (**Figure 3**). A similar phenomenon was observed for predictors developed from the Hoelifch training set in all trials except Tabchy-FEC (data not shown).

**Figure 4:**
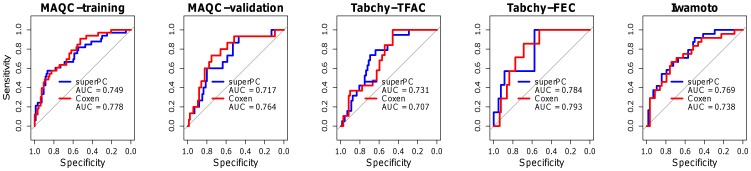
ROC curve validation of MGPs from the Neve training set in 5 clinical trials. In each figure, blue lines represent MGPs developed using the superPC method, while red lines represent MGPs developed using the COXEN method.

We performed receiver operating characteristic (ROC) analysis to evaluate the overall predictability of our MGPs (**Figure 4**). For the TFAC-treated patients, the AUC (Area Under the Curve) values for the superPC and COXEN methods were similar when using either the Neve cohort (0.749, 0.717, 0.731 vs. 0.778, 0.764, 0.707 for MAQC-Training, MAQC-Validation, and Tabchy-TFAC datasets, respectively) or using the Hoeflich database (0.717, 0.733, 0.682 vs. 0.780, 0.746, 0.703 for MAQC-Training, MAQC-Validation, and Tabchy-TFAC datasets, respectively). For the FEC-treated patients, the AUC values for the superPC and COXEN methods were again comparable when using either the Neve database (0.784, 0.769 vs. 0.793, 0.738 for Tabchy-FEC and Iwamoto datasets, respectively) or using the Hoeflich database (0.664, 0.687 vs. 0.688, 0.682 for Tabchy-FEC and Iwamoto datasets, respectively). Overall, these data suggest consistent performance of MGPs derived from the *in vitro* chemoresponse assay on breast cancer cell lines in predicting patient clinical outcome.

We also evaluated superPC and COXEN MGPs by AUC for ER+ and ER– patients separately. For ER+ patients, both the superPC and COXEN models achieved good prediction results for all but the MAQC-validation cohort (**Table 3**). However, for ER– patients, the performances of neither the superPC nor the COXEN models were significant for the test sets, except for the Tabchy-FEC cohort (**Table 3**). Thus, the MGPs developed both with ER+ and ER– breast cancer cells were not predictive for ER– patients, which is consistent with previous studies that reported difficulties in prediction of pCR in ER– cancers [36], [37], [38], [39].

**Table 3:** Prediction results for the superPC and COXEN methods in all breast cancer cell lines evaluated by AUC scores.

			Neve-based prediction		Hoeflich-based prediction	
	Clinical trial test set	ER(+/−)	SuperPC	COXEN	SuperPC	COXEN
**TFAC**	**MAQC-Training**	All	0.749**	0.778**	0.717**	0.780**
		ER+	0.791**	0.732*	0.664*	0.725*
		ER−	0.605	0.564	0.523	0.57
	**MAQC-Validation**	All	0.717**	0.764**	0.733**	0.746**
		ER+	0.449	0.542	0.390	0.424
		ER−	0.524	0.547	0.515	0.577
	**Tabchy-TFAC**	All	0.731**	0.707**	0.682**	0.703**
		ER+	0.750*	0.678	0.728*	0.622
		ER−	0.644*	0.605	0.556	0.615
**FEC**	**Tabchy-FEC**	All	0.784**	0.793**	0.664*	0.688*
		ER+	0.804**	0.819*	0.717	0.587
		ER−	0.902**	0.811**	0.583	0.788*
	**Iwamoto**	All	0.769**	0.738**	0.687**	0.682**
		ER+	0.794**	0.643	0.777*	0.668
		ER−	0.730**	0.706**	0.453	0.539

** : P<0.05,

* : P<0.1.

The AUC values are grouped by ER status: All (cells of both ER status), ER+ (ER− positive cells), and ER– (ER-negative cells) and are separated based on the cell line expression database used to create the cell line MGPs. Note that these five validation datasets (except Tabchy-TFAC and Iwamoto) were independent for the superPC prediction method, because this predictor was not pre-optimized or optimized using any of these data sets. For the COXEN prediction method, MAQC-training and Tabchy-FEC datasets were used for optimization, and therefore the remaining three datasets were truly independent validation sets for this method.

### Evaluation of ER-specific MGPs

In order to improve the prediction for ER– cancers, ER-specific predictors were thus developed using the COXEN model (**Tables S10, S11, S12, S13, S14, S15, S16, S17**). (Due to the small sample size of ER– and ER+ cell lines, an ER-specific predictor-based superPC model did not achieve significant prediction results, data not shown.) The development of ER-specific predictors was similar as before, except that ER– or ER+ predictors were developed separately, based on 22 ER– or 19 ER+ cell lines instead of all 41 cell lines (**Table 4**). The ER– predictors showed a significant improvement in predicting the therapeutic responses and outcomes of ER− patients with AUC values between 0.533 and 0.733 for Neve-based prediction and between 0.503 and 0.818 for Hoeflich-based prediction in all five studies. Thus, the COXEN MGPs specifically developed for the ER– cancers were able to predict pCRs for these patients. By contrast, the ER+-specific predictors performed more poorly for ER+ patients (AUC values between 0.432 and 0.741 for Neve-based prediction and between 0.471 and 0.761 for Hoeflich-based prediction) than the MGPs developed from all cases.

**Table 4:** Prediction results for the COXEN model using either ER+ (ER-positive) or ER– (ER-negative) breast cancer cell lines, evaluated by area under receiver operator characteristic (AU-ROC) scores.

			Neve based prediction		Hoeflich based prediction	
Drug	Clinical trial test set	ER(+/−)	ER+(14)	ER−(16)	ER+(16)	ER−(23)
**TFAC**	**MAQC-Training**	All	0.663**	0.631**	0.443	0.785**
		ER+	0.741*	0.617	0.588	0.676
		ER−	0.644*	0.678**	0.349	0.721**
	**MAQC-Validation**	All	0.599	0.562	0.536	0.677**
		ER+	0.432	0.441	0.508	0.347
		ER−	0.624	0.533	0.536	0.503
	**Tabchy-TFAC**	All	0.582	0.598	0.464	0.774**
		ER+	0.478	0.472	0.594	0.778*
		ER−	0.615	0.699**	0.415	0.726**
**FEC**	**Tabchy-FEC**	All	0.646	0.636	0.504	0.650
		ER+	0.703	0.529	0.761	0.428
		ER−	0.515	0.720	0.295	0.818**
	**Iwamoto**	All	0.647**	0.749**	0.301	0.769**
		ER+	0.735*	0.655	0.471	0.765**
		ER−	0.488	0.733**	0.260	0.706**

** : P<0.05,

* : P<0.1.

The MAQC-Validation and Tabchy-TFAC were truly independent validation sets here because the MAQC-training and Tabchy-FEC were used to optimize the COXEN model.

## Discussion

In this study the prediction performance of MGPs using breast cancer cell lines and an *in vitro* chemoresponse assay was evaluated using different gene expression training sets, multiple test patient sets, two commonly administered chemotherapy combinations, and two different prediction methods. In particular, to create these MGPs we used a large number of breast cancer cell lines both with ER+ and ER– subtypes to represent a wide spectrum of heterogeneous breast tumors. The systematic evaluation performed here showed consistent prediction of patient clinical outcomes by the cell line-derived MGPs across different training sets, two prediction methods, two chemotherapies, and five test patient sets. We believe these results support the potential for using cancer cell line drug sensitivity and genomic data as a proxy for pharmacogenomic data to predict therapeutic responses of breast cancer patients to standard chemotherapies.

Strategies using *in vitro* chemosensitivity data in developing MGPs have a number of advantages over conventional approaches based on patient data. First, the *in vitro* cell line chemoresponse test can efficiently provide molecular biomarker information for the activities of many chemotherapeutic agents, while not relying on complex human patient outcome data. The *in vitro* chemoresponse test can be performed in conjunction with various breast cancer subtypes, e.g. for ER–, HER–, or triple-negative breast cancer (TNBC). This may provide a means to efficiently discover and evaluate effective therapeutic options for these aggressive subtypes of breast cancer, which have been limited due to the relatively small proportions of breast cancer patients exhibiting these subtypes. This strategy also allows one to discover and test biomarkers for various single chemotherapy agents and their combinations, including unusual combinations that have occasionally or never been used in clinics.

Mixed results have been reported in some previous studies of cell line-derived MGPs [12], [14], [15], [16]. While our study demonstrated consistently accurate prediction using *in vitro*-based MGPs, caution is needed in drawing conclusions from this finding, which should be restricted to the chemotherapies investigated in this study. We believe that one of the key issues in using cell lines to develop MGPs is whether the surrogate cell line system can accurately approximate patient clinical outcomes. Gene expression patterns have been found to vary widely between cancer cell lines and patients, possibly diminishing the suitability of cell lines as patient sample surrogates [15], [40]. An artificial *in vitro* system cannot fully mimic a tumor’s *in vivo* environment or emulate complex *in vivo* drug metabolism, and cancer cell lines that can proliferate in artificial *in vitro* environments might represent only a subset of *in vivo* tumor cells [15]. Despite these limitations, we believe that our MGPs have demonstrated potential utility for the following reasons. First, a large number of genes associated with chemosensitivity appear to show expression patterns that are consistent between *in vitro* and *in vivo* environments. Also, while individual genes are highly variable, MGPs aggregating information from multiple genes have the effect of mitigating this variability. A majority of biomarkers from different prediction methods for each chemotherapy treatment shared the same gene networks and functions, such as cellular growth and proliferation, cell morphology, and cell death, despite the relatively small number of overlapping genes (5 biomarkers for TFAC and 2 biomarkers for FEC). As recently reported, we believe there also exist a relatively large number of biomarkers that can effectively stratify tumors with contrasting chemosensitivity [41]. While this research supports the potential of utilizing cell lines for the development of genomic predictors to predict therapeutic responses to chemotherapies, additional development and validation will be necessary to establish the clinical utility of these MGPs.

ER– and ER+ breast cancers have been widely recognized as having heterogeneous gene expression patterns, different mutations, distinct alterations in DNA copy number, and many other differences [42], [43], [44]. ER– cancer patients, in particular, are typically more sensitive to chemotherapy, but often show earlier recurrence and unfavorable prognosis compared to ER+ patients [38], [45], [46]. In this study, we have thus developed and evaluated the MGPs for ER– and ER+ patients separately, which enabled us to accurately predict clinical outcomes for ER– patients. By contrast, past studies have reported difficulties in predicting pCR in ER– patients due to discrepancies in gene expression profile between ER– and ER+ patients [36], [37], [38], [39]. It is unclear why the MGPs developed by exclusively using ER+ cells did not perform well for ER+ patients. It may be due to the small sample size of the subset, with a lower proportion of patients experiencing pCR. This issue will need to be further investigated with more ER+ cell lines and large numbers of patient sets.

## Supporting Information

Table S1 **Summary of chemosensitivity of 27 breast cancer cell lines to FEC and TFAC, and the information of gene expression measured by Neve and Hoeflich.**
(DOC)

Table S2 **MGP-TFAC developed from the Neve training set by the superPC method.**
(DOC)

Table S3 **MGP-FEC developed from the Neve training set by the superPC method.**
(DOC)

Table S4 **MGP-TFAC developed from the Hoeflich training set by the superPC method.**
(DOC)

Table S5 **MGP-FEC developed from the Hoeflich training set by the superPC method.**
(DOC)

Table S6 **MGP-TFAC developed from the Neve training set by the COXEN method.**
(DOC)

Table S7 **MGP-FEC developed from the Neve training set by the COXEN method.**
(DOC)

Table S8 **MGP-TFAC developed from the Hoeflich training set by the COXEN method.**
(DOC)

Table S9 **MGP-FEC developed from the Hoeflich training set by the COXEN method.**
(DOC)

Table S10 **MGP-TFAC developed from the ER positive Neve training set by the COXEN method.**
(DOC)

Table S11 **MGP-TFAC developed from the ER negative Neve training set by the COXEN method.**
(DOC)

Table S12 **MGP-TFAC developed from the ER positive Hoeflich training set by the COXEN method.**
(DOC)

Table S13 **MGP-TFAC developed from the ER negative Hoeflich training set by the COXEN method.**
(DOC)

Table S14 **MGP-FEC developed from the ER positive Neve training set by the COXEN method.**
(DOC)

Table S15 **MGP-FEC developed from the ER negative Neve training set by the COXEN method.**
(DOC)

Table S16 **MGP-FEC developed from the ER positive Hoeflich training set by the COXEN method.**
(DOC)

Table S17 **MGP-FEC developed from the ER negative Hoeflich training set by the COXEN method.**
(DOC)
