# Supplementary material for: A Systematic Evaluation of Multi-Gene Predictors for the Pathological Response of Breast Cancer Patients to Chemotherapy
Source: PLoS One. 2012 Nov 21;7(11):e49529. doi: 10.1371/journal.pone.0049529 (PMC3504014; doi:10.1371/journal.pone.0049529)
Supplement: Table S1 — Summary of chemosensitivity of 27 breast cancer cell lines to FEC and TFAC, and the information of gene expression measured by Neve and Hoeflich. (DOC) [file pone.0049529.s001.doc]

Supplementary Table S1: Summary of chemosensitivity of 27 breast cancer cell lines to FEC and TFAC, measured by ChemoFx, and the information of gene expression measured by Neve and Hoeflich.

| Cell Lines | FEC | TFAC | Neve | Hoeflich |
| --- | --- | --- | --- | --- |
| AU565 | 3.972 | 4.386 | Y | Y |
| BT20 | 6.1 | 6.681 | Y | Y |
| BT474 | 6.76 | 6.558 | Y | Y |
| BT483 | 7.749 | 9.091 | Y | Y |
| BT549 | 3.946 | 4.753 | Y | Y |
| CAL120 | 4.015 | 4.404 |  | Y |
| CAL51 | 4.252 | 4.105 |  | Y |
| CAL851 | 4.294 | 5.141 |  | Y |
| CAMA1 | 5.543 | 6.791 | Y | Y |
| EFM19 | 8.005 | 8.843 |  | Y |
| EFM192A | 6.071 | 7.251 |  | Y |
| EVSAT | 3.824 | 4.302 |  | Y |
| HCC1143 | 5.084 | 5.407 | Y | Y |
| HCC1187 | 4.068 | 4.057 | Y |  |
| HCC1395 | 5.092 | 4.371 |  | Y |
| HCC1419 |  | 8.94 |  | Y |
| HCC1428 | 7.306 | 8.187 | Y | Y |
| HCC1500 | 7.304 | 7.517 | Y | Y |
| HCC1569 |  | 5.675 | Y | Y |
| HCC1806 |  | 3.764 |  | Y |
| HCC1937 | 5.027 | 5.741 | Y | Y |
| HCC1954 | 3.541 | 4.452 | Y | Y |
| HCC38 | 3.591 | 3.729 | Y | Y |
| HDQP1 | 4.967 | 5.106 |  | Y |
| HS578T | 2.813 | 3.367 | Y | Y |
| JIMT1 | 4.592 | 4.45 |  | Y |
| KPL1 | 4.389 | 4.023 |  | Y |
| MCF10A | 4.377 | 4.552 | Y |  |
| MCF7 | 5.193 | 5.813 | Y | Y |
| MDAMB134VI | 5.114 | 5.299 | Y | Y |
| MDAMB157 | 4.361 |  | Y |  |
| MDAMB175VII | 7.804 | 7.905 | Y | Y |
| MDAMB231 | 3.36 | 3.567 | Y | Y |
| MDAMB361 | 7.935 | 8.204 | Y | Y |
| MDAMB415 | 7.154 | 7.196 | Y | Y |
| MDAMB436 | 4.947 | 5.321 | Y | Y |
| MDAMB453 | 6.698 | 6.642 | Y | Y |
| MDAMB468 | 3.077 | 3.578 | Y | Y |
| MFM223 | 4.626 | 4.661 |  | Y |
| SKBR3 | 3.4 | 4.066 | Y | Y |
| SW527 | 4.181 |  |  | Y |
| T47D | 3.534 | 3.86 | Y | Y |
| UACC812 | 2.967 | 3.89 | Y | Y |
| ZR751 | 5.642 | 6.637 | Y | Y |
| ZR7530 | 5.003 | 6.397 | Y | Y |
