# Supplementary material for: A Systematic Evaluation of Multi-Gene Predictors for the Pathological Response of Breast Cancer Patients to Chemotherapy
Source: PLoS One. 2012 Nov 21;7(11):e49529. doi: 10.1371/journal.pone.0049529 (PMC3504014; doi:10.1371/journal.pone.0049529)
Supplement: Table S2 — MGP-TFAC developed from the Neve training set by the superPC method. (DOC) [file pone.0049529.s002.doc]

Supplementary Table S2: MGP-TFAC developed from the Neve training sets by the superPC method.

| Probeset | UniGene.ID | Gene.Symbol | Gene.Title |
| --- | --- | --- | --- |
| 200018_at | Hs.446588 | RPS13 | ribosomal protein S13 |
| 200044_at | Hs.706889 | SFRS9 | splicing factor, arginine/serine-rich 9 |
| 200047_s_at | Hs.388927 | YY1 | YY1 transcription factor |
| 200080_s_at | Hs.533624 | H3F3A /// LOC440926 | H3 histone, family 3A /// H3 histone, family 3A pseudogene |
| 200087_s_at | Hs.75914 | TMED2 | transmembrane emp24 domain trafficking protein 2 |
| 200091_s_at | Hs.512676 | RPS25 | ribosomal protein S25 |
| 200625_s_at | Hs.370581 | CAP1 | CAP, adenylate cyclase-associated protein 1 (yeast) |
| 200794_x_at | Hs.369761 | DAZAP2 | DAZ associated protein 2 |
| 200873_s_at | Hs.125113 | CCT8 | chaperonin containing TCP1, subunit 8 (theta) |
| 200877_at | Hs.421509 | CCT4 | chaperonin containing TCP1, subunit 4 (delta) |
| 200904_at | Hs.650174 | HLA-E | major histocompatibility complex, class I, E |
| 200918_s_at | Hs.368376 | SRPR | sigl recognition particle receptor (docking protein) |
| 200919_at | Hs.524271 | PHC2 | polyhomeotic homolog 2 (Drosophila) |
| 200925_at | Hs.497118 | COX6A1 | cytochrome c oxidase subunit VIa polypeptide 1 |
| 200961_at | Hs.118725 | SEPHS2 | selenophosphate synthetase 2 |
| 200966_x_at | Hs.513490 | ALDOA | aldolase A, fructose-bisphosphate |
| 201089_at | Hs.295917 | ATP6V1B2 | ATPase, H+ transporting, lysosomal 56/58kDa, V1 subunit B2 |
| 201126_s_at | Hs.519818 | MGAT1 | mannosyl (alpha-1,3-)-glycoprotein beta-1,2-N-acetylglucosaminyltransferase |
| 201244_s_at | Hs.159130 | RAF1 | v-raf-1 murine leukemia viral oncogene homolog 1 |
| 201253_s_at | Hs.121549 | CDIPT | CDP-diacylglycerol--inositol 3-phosphatidyltransferase |
| 201276_at | Hs.567328 | RAB5B | RAB5B, member RAS oncogene family |
| 201282_at | Hs.488181 | OGDH | oxoglutarate (alpha-ketoglutarate) dehydrogese (lipoamide) |
| 201292_at | Hs.156346 | TOP2A | topoisomerase (D) II alpha 170kDa |
| 201330_at | Hs.654907 | RARS | arginyl-tR synthetase |
| 201338_x_at | Hs.445977 | GTF3A | general transcription factor IIIA |
| 201509_at | Hs.436405 | IDH3B | isocitrate dehydrogese 3 (D+) beta |
| 201514_s_at | Hs.587054 | G3BP1 | GTPase activating protein (SH3 domain) binding protein 1 |
| 201698_s_at | Hs.706889 | SFRS9 | splicing factor, arginine/serine-rich 9 |
| 201773_at | Hs.570355 | ADNP | activity-dependent neuroprotector homeobox |
| 201853_s_at | Hs.153752 | CDC25B | cell division cycle 25 homolog B (S. pombe) |
| 201863_at | Hs.631614 | FAM32A | family with sequence similarity 32, member A |
| 201871_s_at | Hs.351296 | UBXN1 | UBX domain protein 1 |
| 201970_s_at | Hs.319334 | SP | nuclear autoantigenic sperm protein (histone-binding) |
| 202042_at | Hs.528050 | HARS | histidyl-tR synthetase |
| 202194_at | Hs.482873 | TMED5 | transmembrane emp24 protein transport domain containing 5 |
| 202315_s_at | Hs.517461 | BCR | breakpoint cluster region |
| 202413_s_at | Hs.35086 | USP1 | ubiquitin specific peptidase 1 |
| 202461_at | Hs.409137 | EIF2B2 | eukaryotic translation initiation factor 2B, subunit 2 beta, 39kDa |
| 202521_at | Hs.368367 | CTCF | CCCTC-binding factor (zinc finger protein) |
| 202589_at | Hs.592338 | TYMS | thymidylate synthetase |
| 202680_at | Hs.77100 | GTF2E2 | general transcription factor IIE, polypeptide 2, beta 34kDa |
| 202696_at | Hs.475970 | OXSR1 | oxidative-stress responsive 1 |
| 202752_x_at | Hs.596643 | SLC7A8 | solute carrier family 7 (amino acid transporter, L-type), member 8 |
| 202852_s_at | Hs.254642 | AAGAB | alpha- and gamma-adaptin binding protein |
| 202941_at | Hs.464572 | NDUFV2 | DH dehydrogese (ubiquinone) flavoprotein 2, 24kDa |
| 202956_at | Hs.656902 | ARFGEF1 | ADP-ribosylation factor guanine nucleotide-exchange factor 1(brefeldin A-inhibited) |
| 202985_s_at | Hs.5443 | BAG5 | BCL2-associated athanogene 5 |
| 203311_s_at | Hs.525330 | ARF6 | ADP-ribosylation factor 6 |
| 203554_x_at | Hs.350966 | PTTG1 | pituitary tumor-transforming 1 |
| 204080_at | Hs.525091 | TOE1 | target of EGR1, member 1 (nuclear) |
| 204088_at | Hs.321709 | P2RX4 | purinergic receptor P2X, ligand-gated ion channel, 4 |
| 204355_at | Hs.517948 | DHX30 | DEAH (Asp-Glu-Ala-His) box polypeptide 30 |
| 204812_at | Hs.503886 | ZW10 | ZW10, kinetochore associated, homolog (Drosophila) |
| 206303_s_at | Hs.506325 | NUDT4 | nudix (nucleoside diphosphate linked moiety X)-type motif 4 |
| 207127_s_at | Hs.643472 | HNRNPH3 | heterogeneous nuclear ribonucleoprotein H3 (2H9) |
| 207168_s_at | Hs.420272 | H2AFY | H2A histone family, member Y |
| 207335_x_at | Hs.85539 | ATP5I | ATP synthase, H+ transporting, mitochondrial F0 complex, subunit E |
| 207986_x_at | Hs.355264 | CYB561 | cytochrome b-561 |
| 208024_s_at | Hs.474185 | DGCR6 /// DGCR6L | DiGeorge syndrome critical region gene 6 /// DiGeorge syndrome critical region gene 6-like |
| 208628_s_at | Hs.473583 | YBX1 | Y box binding protein 1 |
| 208737_at | Hs.388654 | ATP6V1G1 | ATPase, H+ transporting, lysosomal 13kDa, V1 subunit G1 |
| 208778_s_at | Hs.363137 | TCP1 | t-complex 1 |
| 208843_s_at | Hs.431317 | GORASP2 | golgi reassembly stacking protein 2, 55kDa |
| 208921_s_at | Hs.489040 | SRI | sorcin |
| 208931_s_at | Hs.465885 | ILF3 | interleukin enhancer binding factor 3, 90kDa |
| 208932_at | Hs.534338 | PPP4C | protein phosphatase 4, catalytic subunit |
| 208971_at | Hs.78601 | UROD | uroporphyrinogen decarboxylase |
| 208974_x_at | Hs.532793 | KPNB1 | karyopherin (importin) beta 1 |
| 208992_s_at | Hs.463059 | STAT3 | sigl transducer and activator of transcription 3 (acute-phase response factor) |
| 209180_at | Hs.78948 | RABGGTB | Rab geranylgeranyltransferase, beta subunit |
| 209181_s_at | Hs.78948 | RABGGTB | Rab geranylgeranyltransferase, beta subunit |
| 209326_at | Hs.21899 | SLC35A2 | solute carrier family 35 (UDP-galactose transporter), member A2 |
| 209408_at | Hs.720061 | KIF2C | kinesin family member 2C |
| 209413_at | Hs.632403 | B4GALT2 | UDP-Gal:betaGlcc beta 1,4- galactosyltransferase, polypeptide 2 |
| 209669_s_at | Hs.724381 | SERBP1 | SERPINE1 mR binding protein 1 |
| 209834_at | Hs.158304 | CHST3 | carbohydrate (chondroitin 6) sulfotransferase 3 |
| 210014_x_at | Hs.436405 | IDH3B | isocitrate dehydrogese 3 (D+) beta |
| 210101_x_at | Hs.136309 | SH3GLB1 | SH3-domain GRB2-like endophilin B1 |
| 210110_x_at | Hs.643472 | HNRNPH3 | heterogeneous nuclear ribonucleoprotein H3 (2H9) |
| 210183_x_at | Hs.409965 | PNN | pinin, desmosome associated protein |
| 210186_s_at | Hs.471933 | FKBP1A | FK506 binding protein 1A, 12kDa |
| 210418_s_at | Hs.436405 | IDH3B | isocitrate dehydrogese 3 (D+) beta |
| 210466_s_at | Hs.724381 | SERBP1 | SERPINE1 mR binding protein 1 |
| 210588_x_at | Hs.643472 | HNRNPH3 | heterogeneous nuclear ribonucleoprotein H3 (2H9) |
| 210825_s_at | Hs.433863 | PEBP1 | phosphatidylethanolamine binding protein 1 |
| 211519_s_at | Hs.720061 | KIF2C | kinesin family member 2C |
| 211686_s_at | Hs.583805 | MAK16 | MAK16 homolog (S. cerevisiae) |
| 211934_x_at | Hs.595071 | GAB | glucosidase, alpha; neutral AB |
| 211940_x_at | Hs.533624 | H3F3A /// LOC440926 | H3 histone, family 3A /// H3 histone, family 3A pseudogene |
| 211954_s_at | Hs.712598 | IPO5 | importin 5 |
| 211955_at | Hs.712598 | IPO5 | importin 5 |
| 211961_s_at | Hs.723832 | RAB7A | RAB7A, member RAS oncogene family |
| 212031_at | Hs.531106 | RBM25 | R binding motif protein 25 |
| 212082_s_at | Hs.632717 | MYL6 | myosin, light chain 6, alkali, smooth muscle and non-muscle |
| 212137_at | Hs.292078 | LARP1 | La ribonucleoprotein domain family, member 1 |
| 212155_at | Hs.356377 | RNF187 | ring finger protein 187 |
| 212166_at | Hs.172685 | XPO7 | exportin 7 |
| 212193_s_at | Hs.292078 | LARP1 | La ribonucleoprotein domain family, member 1 |
| 212204_at | Hs.724532 | TMEM87A | transmembrane protein 87A |
| 212231_at | Hs.159699 | FBXO21 | F-box protein 21 |
| 212378_at | Hs.473648 | GART | phosphoribosylglycimide formyltransferase, phosphoribosylglycimide synthetase, phosphoribosylaminoimidazole synthetase |
| 212400_at | Hs.535972 | FAM102A | family with sequence similarity 102, member A |
| 212465_at | Hs.510407 | SETD3 | SET domain containing 3 |
| 212506_at | Hs.163893 | PICALM | phosphatidylinositol binding clathrin assembly protein |
| 212507_at | Hs.469376 | TMEM131 | transmembrane protein 131 |
| 212519_at | Hs.164853 | UBE2E1 | ubiquitin-conjugating enzyme E2E 1 (UBC4/5 homolog, yeast) |
| 212528_at | Hs.633087 | --- | --- |
| 212692_s_at | Hs.480938 | LRBA | LPS-responsive vesicle trafficking, beach and anchor containing |
| 212833_at | Hs.75639 | SLC25A46 | solute carrier family 25, member 46 |
| 213574_s_at | Hs.595245 | --- | --- |
| 213798_s_at | Hs.370581 | CAP1 | CAP, adenylate cyclase-associated protein 1 (yeast) |
| 214687_x_at | Hs.513490 | ALDOA | aldolase A, fructose-bisphosphate |
| 215493_x_at | Hs.159028 | BTN2A1 | butyrophilin, subfamily 2, member A1 |
| 215696_s_at | Hs.668588 | SEC16A | SEC16 homolog A (S. cerevisiae) |
| 216092_s_at | Hs.596643 | SLC7A8 | solute carrier family 7 (amino acid transporter, L-type), member 8 |
| 217200_x_at | Hs.355264 | CYB561 | cytochrome b-561 |
| 217724_at | Hs.724381 | SERBP1 | SERPINE1 mR binding protein 1 |
| 217725_x_at | Hs.724381 | SERBP1 | SERPINE1 mR binding protein 1 |
| 217756_x_at | Hs.424126 | SERF2 | small EDRK-rich factor 2 |
| 217844_at | Hs.444468 | CTDSP1 | CTD (carboxy-termil domain, R polymerase II, polypeptide A) small phosphatase 1 |
| 217942_at | Hs.311072 | MRPS35 | mitochondrial ribosomal protein S35 |
| 217971_at | Hs.723128 | MAPKSP1 | MAPK scaffold protein 1 |
| 218010_x_at | Hs.79625 | PPDPF | pancreatic progenitor cell differentiation and proliferation factor homolog (zebrafish) |
| 218028_at | Hs.25597 | ELOVL1 | elongation of very long chain fatty acids (FEN1/Elo2, SUR4/Elo3, yeast)-like 1 |
| 218080_x_at | Hs.530402 | FAF1 | Fas (TNFRSF6) associated factor 1 |
| 218138_at | Hs.472119 | MKKS | McKusick-Kaufman syndrome |
| 218226_s_at | Hs.304613 | NDUFB4 | DH dehydrogese (ubiquinone) 1 beta subcomplex, 4, 15kDa |
| 218245_at | Hs.8361 | TSKU | tsukushi small leucine rich proteoglycan homolog (Xenopus laevis) |
| 218250_s_at | Hs.598387 | CNOT7 | CCR4-NOT transcription complex, subunit 7 |
| 218259_at | Hs.49143 | MKL2 | MKL/myocardin-like 2 |
| 218320_s_at | Hs.521969 | NDUFB11 | DH dehydrogese (ubiquinone) 1 beta subcomplex, 11, 17.3kDa |
| 218384_at | Hs.632184 | CARHSP1 | calcium regulated heat stable protein 1, 24kDa |
| 218448_at | Hs.353013 | C20orf11 | chromosome 20 open reading frame 11 |
| 218481_at | Hs.283741 | EXOSC5 | exosome component 5 |
| 218494_s_at | Hs.435126 | SLC2A4RG | SLC2A4 regulator |
| 218496_at | Hs.568006 | RSEH1 | ribonuclease H1 |
| 218558_s_at | Hs.420696 | MRPL39 | mitochondrial ribosomal protein L39 |
| 219076_s_at | Hs.430299 | PXMP2 | peroxisomal membrane protein 2, 22kDa |
| 219220_x_at | Hs.75724 | MRPS22 | mitochondrial ribosomal protein S22 |
| 219649_at | Hs.258501 | ALG6 | asparagine-linked glycosylation 6, alpha-1,3-glucosyltransferase homolog (S. cerevisiae) |
| 220964_s_at | Hs.300816 | RAB1B | RAB1B, member RAS oncogene family |
| 221263_s_at | Hs.110695 | SF3B5 | splicing factor 3b, subunit 5, 10kDa |
| 221520_s_at | Hs.524571 | CDCA8 | cell division cycle associated 8 |
| 221637_s_at | Hs.9061 | C11orf48 | chromosome 11 open reading frame 48 |
| 221649_s_at | Hs.14468 | PPAN | peter pan homolog (Drosophila) |
| 221691_x_at | Hs.557550 | NPM1 | nucleophosmin (nucleolar phosphoprotein B23, numatrin) |
| 221712_s_at | Hs.724467 | WDR74 | WD repeat domain 74 |
| 221856_s_at | Hs.723127 | FAM63A | family with sequence similarity 63, member A |
| 221923_s_at | Hs.557550 | NPM1 | nucleophosmin (nucleolar phosphoprotein B23, numatrin) |
| 46256_at | Hs.592080 | SPSB3 | splA/ryanodine receptor domain and SOCS box containing 3 |
