# Supplementary material for: A Systematic Evaluation of Multi-Gene Predictors for the Pathological Response of Breast Cancer Patients to Chemotherapy
Source: PLoS One. 2012 Nov 21;7(11):e49529. doi: 10.1371/journal.pone.0049529 (PMC3504014; doi:10.1371/journal.pone.0049529)
Supplement: Table S4 — MGP-TFAC developed from the Hoeflich training set by the superPC method. (DOC) [file pone.0049529.s004.doc]

Supplementary Table S4: MGP-TFAC developed from the Hoeflich training sets by the superPC method.

| Probeset | UniGene.ID | Gene.Symbol | Gene.Title |
| --- | --- | --- | --- |
| 200054_at | Hs.7165 | ZNF259 | zinc finger protein 259 |
| 200074_s_at | Hs.446522 | RPL14 | ribosomal protein L14 |
| 200749_at | --- | --- | --- |
| 200804_at | Hs.708025 | TMBIM6 | transmembrane BAX inhibitor motif containing 6 |
| 200895_s_at | Hs.524183 | FKBP4 | FK506 binding protein 4, 59kDa |
| 200925_at | Hs.497118 | COX6A1 | cytochrome c oxidase subunit VIa polypeptide 1 |
| 200941_at | Hs.250899 | HSBP1 | heat shock factor binding protein 1 |
| 201064_s_at | Hs.169900 | PABPC4 | poly(A) binding protein, cytoplasmic 4 (inducible form) |
| 201129_at | Hs.309090 | SFRS7 | splicing factor, arginine/serine-rich 7, 35kDa |
| 201174_s_at | Hs.710553 | TERF2IP | telomeric repeat binding factor 2, interacting protein |
| 201176_s_at | Hs.33642 | ARCN1 | archain 1 |
| 201231_s_at | Hs.517145 | ENO1 | enolase 1, (alpha) |
| 201276_at | Hs.567328 | RAB5B | RAB5B, member RAS oncogene family |
| 201371_s_at | Hs.372286 | CUL3 | cullin 3 |
| 201443_s_at | Hs.495960 | ATP6AP2 | ATPase, H+ transporting, lysosomal accessory protein 2 |
| 201499_s_at | Hs.386939 | USP7 | ubiquitin specific peptidase 7 (herpes virus-associated) |
| 201503_at | Hs.587054 | G3BP1 | GTPase activating protein (SH3 domain) binding protein 1 |
| 201623_s_at | Hs.503787 | DARS | aspartyl-tR synthetase |
| 201712_s_at | Hs.199561 | RANBP2 | RAN binding protein 2 |
| 201972_at | Hs.477155 | ATP6V1A | ATPase, H+ transporting, lysosomal 70kDa, V1 subunit A |
| 202026_at | Hs.356270 | SDHD | succite dehydrogese complex, subunit D, integral membrane protein |
| 202076_at | Hs.696238 | BIRC2 | baculoviral IAP repeat-containing 2 |
| 202106_at | Hs.507333 | GOLGA3 | golgin A3 |
| 202137_s_at | Hs.292265 | ZMYND11 | zinc finger, MYND domain containing 11 |
| 202144_s_at | Hs.75527 | ADSL | adenylosuccite lyase |
| 202170_s_at | Hs.524009 | AASDHPPT | aminoadipate-semialdehyde dehydrogese-phosphopantetheinyl transferase |
| 202302_s_at | Hs.432996 | RSRC2 | arginine/serine-rich coiled-coil 2 |
| 202346_at | Hs.724519 | UBE2K | ubiquitin-conjugating enzyme E2K (UBC1 homolog, yeast) |
| 202385_s_at | Hs.519672 | TCOF1 | Treacher Collins-Franceschetti syndrome 1 |
| 202521_at | Hs.368367 | CTCF | CCCTC-binding factor (zinc finger protein) |
| 203040_s_at | Hs.82609 | HMBS | hydroxymethylbilane synthase |
| 203089_s_at | Hs.724371 | HTRA2 | HtrA serine peptidase 2 |
| 203119_at | Hs.4253 | CCDC86 | coiled-coil domain containing 86 |
| 203341_at | Hs.135406 | CEBPZ | CCAAT/enhancer binding protein (C/EBP), zeta |
| 203383_s_at | Hs.133469 | GOLGA1 | golgin A1 |
| 203405_at | Hs.473838 | PSMG1 | proteasome (prosome, macropain) assembly chaperone 1 |
| 203492_x_at | Hs.101014 | CEP57 | centrosomal protein 57kDa |
| 203614_at | --- | UTP14C | UTP14, U3 small nucleolar ribonucleoprotein, homolog C (yeast) |
| 203707_at | Hs.611475 | ZNF263 | zinc finger protein 263 |
| 203825_at | Hs.522472 | BRD3 | bromodomain containing 3 |
| 203831_at | Hs.443673 | R3HDM2 | R3H domain containing 2 |
| 203870_at | Hs.7966 | USP46 | ubiquitin specific peptidase 46 |
| 203944_x_at | Hs.159028 | BTN2A1 | butyrophilin, subfamily 2, member A1 |
| 204144_s_at | Hs.644153 | PIGQ | phosphatidylinositol glycan anchor biosynthesis, class Q |
| 204251_s_at | Hs.504009 | CEP164 | centrosomal protein 164kDa |
| 204315_s_at | Hs.386189 | GTSE1 | G-2 and S-phase expressed 1 |
| 204327_s_at | Hs.112556 | ZNF202 | zinc finger protein 202 |
| 204371_s_at | Hs.723206 | KHSRP | KH-type splicing regulatory protein |
| 204690_at | Hs.431109 | STX8 | syntaxin 8 |
| 204977_at | Hs.591931 | DDX10 | DEAD (Asp-Glu-Ala-Asp) box polypeptide 10 |
| 205012_s_at | Hs.157394 | HAGH | hydroxyacylglutathione hydrolase |
| 205176_s_at | Hs.166539 | ITGB3BP | integrin beta 3 binding protein (beta3-endonexin) |
| 205202_at | Hs.279257 | PCMT1 | protein-L-isoaspartate (D-aspartate) O-methyltransferase |
| 205203_at | Hs.382865 | PLD1 | phospholipase D1, phosphatidylcholine-specific |
| 205252_at | Hs.155204 | ZNF174 | zinc finger protein 174 |
| 206098_at | Hs.654596 | ZBTB6 | zinc finger and BTB domain containing 6 |
| 207112_s_at | Hs.618456 | GAB1 | GRB2-associated binding protein 1 |
| 207458_at | Hs.245886 | C8orf51 | chromosome 8 open reading frame 51 |
| 207573_x_at | Hs.486360 | ATP5L | ATP synthase, H+ transporting, mitochondrial F0 complex, subunit G |
| 207941_s_at | Hs.282901 | RBM39 | R binding motif protein 39 |
| 208398_s_at | Hs.486507 | TBPL1 | TBP-like 1 |
| 208627_s_at | Hs.473583 | YBX1 | Y box binding protein 1 |
| 208636_at | Hs.509765 | ACTN1 | actinin, alpha 1 |
| 208736_at | Hs.524741 | ARPC3 | actin related protein 2/3 complex, subunit 3, 21kDa |
| 208737_at | Hs.388654 | ATP6V1G1 | ATPase, H+ transporting, lysosomal 13kDa, V1 subunit G1 |
| 208746_x_at | Hs.486360 | ATP5L | ATP synthase, H+ transporting, mitochondrial F0 complex, subunit G |
| 208756_at | Hs.530096 | EIF3I | eukaryotic translation initiation factor 3, subunit I |
| 209575_at | Hs.654593 | IL10RB | interleukin 10 receptor, beta |
| 209630_s_at | Hs.494985 | FBXW2 | F-box and WD repeat domain containing 2 |
| 209798_at | Hs.171061 | NPAT | nuclear protein, ataxia-telangiectasia locus |
| 209862_s_at | Hs.101014 | CEP57 | centrosomal protein 57kDa |
| 210005_at | Hs.473648 | GART | phosphoribosylglycimide formyltransferase, phosphoribosylglycimide synthetase, phosphoribosylaminoimidazole synthetase |
| 210160_at | Hs.724435 | PAFAH1B2 | platelet-activating factor acetylhydrolase 1b, catalytic subunit 2 (30kDa) |
| 210183_x_at | Hs.409965 | PNN | pinin, desmosome associated protein |
| 210250_x_at | Hs.75527 | ADSL | adenylosuccite lyase |
| 210453_x_at | Hs.486360 | ATP5L | ATP synthase, H+ transporting, mitochondrial F0 complex, subunit G |
| 210466_s_at | Hs.724381 | SERBP1 | SERPINE1 mR binding protein 1 |
| 211034_s_at | Hs.695995 | C12orf51 | chromosome 12 open reading frame 51 |
| 211392_s_at | Hs.517557 | PATZ1 | POZ (BTB) and AT hook containing zinc finger 1 |
| 211584_s_at | Hs.171061 | NPAT | nuclear protein, ataxia-telangiectasia locus |
| 211749_s_at | Hs.66708 | VAMP3 | vesicle-associated membrane protein 3 (cellubrevin) |
| 212064_x_at | Hs.23650 | MAZ | MYC-associated zinc finger protein (purine-binding transcription factor) |
| 212331_at | Hs.513609 | RBL2 | retinoblastoma-like 2 (p130) |
| 212367_at | Hs.362733 | FEM1B | fem-1 homolog b (C. elegans) |
| 212400_at | Hs.535972 | FAM102A | family with sequence similarity 102, member A |
| 212403_at | Hs.374067 | UBE3B | ubiquitin protein ligase E3B |
| 212506_at | Hs.163893 | PICALM | phosphatidylinositol binding clathrin assembly protein |
| 212547_at | Hs.522472 | BRD3 | bromodomain containing 3 |
| 212871_at | Hs.413901 | MAPKAPK5 | mitogen-activated protein kise-activated protein kise 5 |
| 212920_at | Hs.631513 | REST | RE1-silencing transcription factor |
| 213141_at | Hs.513683 | PSKH1 | protein serine kise H1 |
| 213185_at | Hs.723969 | KIAA0556 | KIAA0556 |
| 213196_at | Hs.301094 | ZNF629 | zinc finger protein 629 |
| 213473_at | Hs.530940 | BRAP | BRCA1 associated protein |
| 213681_at | Hs.459379 | CYHR1 | cysteine/histidine-rich 1 |
| 213743_at | Hs.591241 | CCNT2 | cyclin T2 |
| 213798_s_at | Hs.370581 | CAP1 | CAP, adenylate cyclase-associated protein 1 (yeast) |
| 213907_at | Hs.602353 | EEF1E1 | Eukaryotic translation elongation factor 1 epsilon 1 |
| 214138_at | Hs.522399 | ZNF79 | zinc finger protein 79 |
| 214483_s_at | Hs.416089 | ARFIP1 | ADP-ribosylation factor interacting protein 1 |
| 214635_at | Hs.296949 | CLDN9 | claudin 9 |
| 215493_x_at | Hs.159028 | BTN2A1 | butyrophilin, subfamily 2, member A1 |
| 215696_s_at | Hs.668588 | SEC16A | SEC16 homolog A (S. cerevisiae) |
| 216389_s_at | Hs.525251 | DCAF11 | DDB1 and CUL4 associated factor 11 |
| 216624_s_at | Hs.258855 | MLL | myeloid/lymphoid or mixed-lineage leukemia (trithorax homolog, Drosophila) |
| 217156_at | --- | --- | --- |
| 217294_s_at | Hs.517145 | ENO1 | enolase 1, (alpha) |
| 217445_s_at | Hs.473648 | GART | phosphoribosylglycimide formyltransferase, phosphoribosylglycimide synthetase, phosphoribosylaminoimidazole synthetase |
| 217747_s_at | Hs.546288 | RPS9 | ribosomal protein S9 |
| 217756_x_at | Hs.424126 | SERF2 | small EDRK-rich factor 2 |
| 217777_s_at | Hs.512973 | PTPLAD1 | protein tyrosine phosphatase-like A domain containing 1 |
| 217795_s_at | Hs.517817 | TMEM43 | transmembrane protein 43 |
| 217939_s_at | Hs.655167 | AFTPH | aftiphilin |
| 218107_at | Hs.497873 | WDR26 | WD repeat domain 26 |
| 218333_at | Hs.286131 | DERL2 | Der1-like domain family, member 2 |
| 218494_s_at | Hs.435126 | SLC2A4RG | SLC2A4 regulator |
| 218527_at | Hs.20158 | APTX | aprataxin |
| 218533_s_at | Hs.504998 | UCKL1 | uridine-cytidine kise 1-like 1 |
| 218566_s_at | Hs.22857 | CHORDC1 | cysteine and histidine-rich domain (CHORD)-containing 1 |
| 218696_at | Hs.591589 | EIF2AK3 | eukaryotic translation initiation factor 2-alpha kise 3 |
| 218754_at | Hs.59425 | NOL9 | nucleolar protein 9 |
| 219023_at | Hs.435991 | AP1AR | adaptor-related protein complex 1 associated regulatory protein |
| 219098_at | Hs.22824 | MYBBP1A | MYB binding protein (P160) 1a |
| 219122_s_at | Hs.353090 | THG1L | tR-histidine guanylyltransferase 1-like (S. cerevisiae) |
| 219223_at | Hs.62003 | C9orf7 | chromosome 9 open reading frame 7 |
| 219679_s_at | Hs.435610 | WAC | WW domain containing adaptor with coiled-coil |
| 219979_s_at | Hs.283322 | C11orf73 | chromosome 11 open reading frame 73 |
| 220223_at | Hs.528902 | ATAD5 | ATPase family, AAA domain containing 5 |
| 220606_s_at | Hs.47668 | C17orf48 | chromosome 17 open reading frame 48 |
| 221230_s_at | Hs.575782 | ARID4B | AT rich interactive domain 4B (RBP1-like) |
| 221253_s_at | Hs.150837 | MUTED /// TXNDC5 | muted homolog (mouse) /// thioredoxin domain containing 5 (endoplasmic reticulum) |
| 221434_s_at | Hs.655105 | C14orf156 | chromosome 14 open reading frame 156 |
| 221517_s_at | Hs.444931 | MED17 | mediator complex subunit 17 |
| 221580_s_at | Hs.355750 | TAF1D | TATA box binding protein (TBP)-associated factor, R polymerase I, D, 41kDa |
| 221691_x_at | Hs.557550 | NPM1 | nucleophosmin (nucleolar phosphoprotein B23, numatrin) |
| 221769_at | Hs.592080 | SPSB3 | splA/ryanodine receptor domain and SOCS box containing 3 |
| 221836_s_at | Hs.654911 | TRAPPC9 | trafficking protein particle complex 9 |
| 221923_s_at | Hs.557550 | NPM1 | nucleophosmin (nucleolar phosphoprotein B23, numatrin) |
| 222000_at | Hs.103939 | C1orf174 | chromosome 1 open reading frame 174 |
| 32029_at | Hs.459691 | PDPK1 | 3-phosphoinositide dependent protein kise-1 |
| 33307_at | Hs.360940 | RRP7A | ribosomal R processing 7 homolog A (S. cerevisiae) |
| 35436_at | Hs.155827 | GOLGA2 | golgin A2 |
| 41512_at | Hs.530940 | BRAP | BRCA1 associated protein |
| 45526_g_at | Hs.513296 | T15 | N-acetyltransferase 15 (GCN5-related, putative) |
| 46256_at | Hs.592080 | SPSB3 | splA/ryanodine receptor domain and SOCS box containing 3 |
| 49485_at | Hs.506655 | PRDM4 | PR domain containing 4 |
| 50376_at | Hs.24545 | ZNF444 | zinc finger protein 444 |
| 56829_at | Hs.654911 | TRAPPC9 | trafficking protein particle complex 9 |
| 61874_at | Hs.62003 | C9orf7 | chromosome 9 open reading frame 7 |
| 77508_r_at | Hs.555978 | RABEP2 | rabaptin, RAB GTPase binding effector protein 2 |
