# Supplementary material for: A Systematic Evaluation of Multi-Gene Predictors for the Pathological Response of Breast Cancer Patients to Chemotherapy
Source: PLoS One. 2012 Nov 21;7(11):e49529. doi: 10.1371/journal.pone.0049529 (PMC3504014; doi:10.1371/journal.pone.0049529)
Supplement: Table S5 — MGP-FEC developed from the Hoeflich training set by the superPC method. (DOC) [file pone.0049529.s005.doc]

Supplementary Table S5: MGP-FEC developed from the Hoeflich training sets by the superPC method.

| Probeset | UniGene.ID | Gene.Symbol | Gene.Title |
| --- | --- | --- | --- |
| 200044_at | Hs.706889 | SFRS9 | splicing factor, arginine/serine-rich 9 |
| 200054_at | Hs.7165 | ZNF259 | zinc finger protein 259 |
| 200069_at | Hs.584842 | SART3 | squamous cell carcinoma antigen recognized by T cells 3 |
| 200074_s_at | Hs.446522 | RPL14 | ribosomal protein L14 |
| 200617_at | Hs.724480 | MLEC | malectin |
| 200804_at | Hs.708025 | TMBIM6 | transmembrane BAX inhibitor motif containing 6 |
| 200836_s_at | Hs.517949 | MAP4 | microtubule-associated protein 4 |
| 200861_at | Hs.724510 | CNOT1 | CCR4-NOT transcription complex, subunit 1 |
| 200864_s_at | Hs.321541 | RAB11A | RAB11A, member RAS oncogene family |
| 200925_at | Hs.497118 | COX6A1 | cytochrome c oxidase subunit VIa polypeptide 1 |
| 200941_at | Hs.250899 | HSBP1 | heat shock factor binding protein 1 |
| 201174_s_at | Hs.710553 | TERF2IP | telomeric repeat binding factor 2, interacting protein |
| 201176_s_at | Hs.33642 | ARCN1 | archain 1 |
| 201216_at | Hs.75841 | ERP29 | endoplasmic reticulum protein 29 |
| 201231_s_at | Hs.517145 | ENO1 | enolase 1, (alpha) |
| 201276_at | Hs.567328 | RAB5B | RAB5B, member RAS oncogene family |
| 201336_at | Hs.66708 | VAMP3 | vesicle-associated membrane protein 3 (cellubrevin) |
| 201337_s_at | Hs.66708 | VAMP3 | vesicle-associated membrane protein 3 (cellubrevin) |
| 201370_s_at | Hs.372286 | CUL3 | cullin 3 |
| 201371_s_at | Hs.372286 | CUL3 | cullin 3 |
| 201499_s_at | Hs.386939 | USP7 | ubiquitin specific peptidase 7 (herpes virus-associated) |
| 201503_at | Hs.587054 | G3BP1 | GTPase activating protein (SH3 domain) binding protein 1 |
| 201609_x_at | Hs.515688 | ICMT | isoprenylcysteine carboxyl methyltransferase |
| 201698_s_at | Hs.706889 | SFRS9 | splicing factor, arginine/serine-rich 9 |
| 201716_at | Hs.188634 | SNX1 | sorting nexin 1 |
| 201948_at | Hs.75528 | GNL2 | guanine nucleotide binding protein-like 2 (nucleolar) |
| 201990_s_at | Hs.591156 | CREBL2 | cAMP responsive element binding protein-like 2 |
| 202042_at | Hs.528050 | HARS | histidyl-tR synthetase |
| 202076_at | Hs.696238 | BIRC2 | baculoviral IAP repeat-containing 2 |
| 202106_at | Hs.507333 | GOLGA3 | golgin A3 |
| 202136_at | Hs.292265 | ZMYND11 | zinc finger, MYND domain containing 11 |
| 202137_s_at | Hs.292265 | ZMYND11 | zinc finger, MYND domain containing 11 |
| 202296_s_at | Hs.724419 | RER1 | RER1 retention in endoplasmic reticulum 1 homolog (S. cerevisiae) |
| 202302_s_at | Hs.432996 | RSRC2 | arginine/serine-rich coiled-coil 2 |
| 202521_at | Hs.368367 | CTCF | CCCTC-binding factor (zinc finger protein) |
| 202696_at | Hs.475970 | OXSR1 | oxidative-stress responsive 1 |
| 202774_s_at | Hs.308171 | SFRS8 | splicing factor, arginine/serine-rich 8 (suppressor-of-white-apricot homolog, Drosophila) |
| 202852_s_at | Hs.254642 | AAGAB | alpha- and gamma-adaptin binding protein |
| 203341_at | Hs.135406 | CEBPZ | CCAAT/enhancer binding protein (C/EBP), zeta |
| 203693_s_at | Hs.703174 | E2F3 | E2F transcription factor 3 |
| 203825_at | Hs.522472 | BRD3 | bromodomain containing 3 |
| 203944_x_at | Hs.159028 | BTN2A1 | butyrophilin, subfamily 2, member A1 |
| 204251_s_at | Hs.504009 | CEP164 | centrosomal protein 164kDa |
| 204523_at | Hs.181552 | ZNF140 | zinc finger protein 140 |
| 204690_at | Hs.431109 | STX8 | syntaxin 8 |
| 204977_at | Hs.591931 | DDX10 | DEAD (Asp-Glu-Ala-Asp) box polypeptide 10 |
| 205012_s_at | Hs.157394 | HAGH | hydroxyacylglutathione hydrolase |
| 205252_at | Hs.155204 | ZNF174 | zinc finger protein 174 |
| 206098_at | Hs.654596 | ZBTB6 | zinc finger and BTB domain containing 6 |
| 207458_at | Hs.245886 | C8orf51 | chromosome 8 open reading frame 51 |
| 207573_x_at | Hs.486360 | ATP5L | ATP synthase, H+ transporting, mitochondrial F0 complex, subunit G |
| 207941_s_at | Hs.282901 | RBM39 | R binding motif protein 39 |
| 208627_s_at | Hs.473583 | YBX1 | Y box binding protein 1 |
| 208654_s_at | Hs.520313 | CD164 | CD164 molecule, sialomucin |
| 208737_at | Hs.388654 | ATP6V1G1 | ATPase, H+ transporting, lysosomal 13kDa, V1 subunit G1 |
| 208746_x_at | Hs.486360 | ATP5L | ATP synthase, H+ transporting, mitochondrial F0 complex, subunit G |
| 208756_at | Hs.530096 | EIF3I | eukaryotic translation initiation factor 3, subunit I |
| 208874_x_at | Hs.400740 | PPP2R4 | protein phosphatase 2A activator, regulatory subunit 4 |
| 209112_at | Hs.238990 | CDKN1B | cyclin-dependent kise inhibitor 1B (p27, Kip1) |
| 209196_at | Hs.520063 | WDR46 | WD repeat domain 46 |
| 209202_s_at | Hs.491354 | EXTL3 | exostoses (multiple)-like 3 |
| 209390_at | Hs.370854 | TSC1 | tuberous sclerosis 1 |
| 209431_s_at | Hs.517557 | PATZ1 | POZ (BTB) and AT hook containing zinc finger 1 |
| 209630_s_at | Hs.494985 | FBXW2 | F-box and WD repeat domain containing 2 |
| 209669_s_at | Hs.724381 | SERBP1 | SERPINE1 mR binding protein 1 |
| 209798_at | Hs.171061 | NPAT | nuclear protein, ataxia-telangiectasia locus |
| 209934_s_at | Hs.584884 | ATP2C1 | ATPase, Ca++ transporting, type 2C, member 1 |
| 210097_s_at | Hs.306242 | NOL7 | nucleolar protein 7, 27kDa |
| 210453_x_at | Hs.486360 | ATP5L | ATP synthase, H+ transporting, mitochondrial F0 complex, subunit G |
| 210466_s_at | Hs.724381 | SERBP1 | SERPINE1 mR binding protein 1 |
| 210555_s_at | Hs.632209 | NFATC3 | nuclear factor of activated T-cells, cytoplasmic, calcineurin-dependent 3 |
| 210581_x_at | Hs.517557 | PATZ1 | POZ (BTB) and AT hook containing zinc finger 1 |
| 211150_s_at | Hs.335551 | DLAT | dihydrolipoamide S-acetyltransferase |
| 211391_s_at | Hs.517557 | PATZ1 | POZ (BTB) and AT hook containing zinc finger 1 |
| 211392_s_at | Hs.517557 | PATZ1 | POZ (BTB) and AT hook containing zinc finger 1 |
| 211749_s_at | Hs.66708 | VAMP3 | vesicle-associated membrane protein 3 (cellubrevin) |
| 212064_x_at | Hs.23650 | MAZ | MYC-associated zinc finger protein (purine-binding transcription factor) |
| 212068_s_at | Hs.724374 | BAT2L1 | HLA-B associated transcript 2-like 1 |
| 212229_s_at | Hs.159699 | FBXO21 | F-box protein 21 |
| 212252_at | Hs.297343 | CAMKK2 | calcium/calmodulin-dependent protein kise kise 2, beta |
| 212367_at | Hs.362733 | FEM1B | fem-1 homolog b (C. elegans) |
| 212383_at | Hs.463074 | ATP6V0A1 | ATPase, H+ transporting, lysosomal V0 subunit a1 |
| 212403_at | Hs.374067 | UBE3B | ubiquitin protein ligase E3B |
| 212452_x_at | Hs.35758 | MYST4 | MYST histone acetyltransferase (monocytic leukemia) 4 |
| 212547_at | Hs.522472 | BRD3 | bromodomain containing 3 |
| 212571_at | Hs.530698 | CHD8 | chromodomain helicase D binding protein 8 |
| 212995_x_at | Hs.469925 | FAM128B | family with sequence similarity 128, member B |
| 213025_at | Hs.460232 | THUMPD1 | THUMP domain containing 1 |
| 213055_at | Hs.446414 | CD47 | CD47 molecule |
| 213141_at | Hs.513683 | PSKH1 | protein serine kise H1 |
| 213185_at | Hs.723969 | KIAA0556 | KIAA0556 |
| 213196_at | Hs.301094 | ZNF629 | zinc finger protein 629 |
| 213234_at | Hs.132660 | KIAA1467 | KIAA1467 |
| 213473_at | Hs.530940 | BRAP | BRCA1 associated protein |
| 213588_x_at | Hs.446522 | RPL14 | ribosomal protein L14 |
| 213615_at | Hs.655248 | LPCAT3 | Lysophosphatidylcholine acyltransferase 3 |
| 213743_at | Hs.591241 | CCNT2 | cyclin T2 |
| 213798_s_at | Hs.370581 | CAP1 | CAP, adenylate cyclase-associated protein 1 (yeast) |
| 213907_at | Hs.602353 | EEF1E1 | Eukaryotic translation elongation factor 1 epsilon 1 |
| 214483_s_at | Hs.416089 | ARFIP1 | ADP-ribosylation factor interacting protein 1 |
| 214635_at | Hs.296949 | CLDN9 | claudin 9 |
| 215458_s_at | Hs.189329 | SMURF1 | SMAD specific E3 ubiquitin protein ligase 1 |
| 215493_x_at | Hs.159028 | BTN2A1 | butyrophilin, subfamily 2, member A1 |
| 216226_at | Hs.369519 | TAF4B | TAF4b R polymerase II, TATA box binding protein (TBP)-associated factor, 105kDa |
| 216389_s_at | Hs.525251 | DCAF11 | DDB1 and CUL4 associated factor 11 |
| 217156_at | --- | --- | --- |
| 217185_s_at | Hs.7165 | ZNF259 /// ZNF259P1 | zinc finger protein 259 /// zinc finger protein 259 pseudogene 1 |
| 217294_s_at | Hs.517145 | ENO1 | enolase 1, (alpha) |
| 217722_s_at | Hs.135471 | NGRN | neugrin, neurite outgrowth associated |
| 217777_s_at | Hs.512973 | PTPLAD1 | protein tyrosine phosphatase-like A domain containing 1 |
| 217860_at | Hs.277677 | LOC732160 /// NDUFA10 | similar to DH dehydrogese (ubiquinone) 1 alpha subcomplex, 10, 42kDa precursor /// DH dehydrogese (ubiquinone) 1 alpha subcomplex, 10, 42kDa |
| 217939_s_at | Hs.655167 | AFTPH | aftiphilin |
| 217942_at | Hs.311072 | MRPS35 | mitochondrial ribosomal protein S35 |
| 217994_x_at | Hs.6449 | CPSF3L | cleavage and polyadenylation specific factor 3-like |
| 218135_at | Hs.339453 | ERGIC2 | ERGIC and golgi 2 |
| 218259_at | Hs.49143 | MKL2 | MKL/myocardin-like 2 |
| 218333_at | Hs.286131 | DERL2 | Der1-like domain family, member 2 |
| 218488_at | Hs.533549 | EIF2B3 | eukaryotic translation initiation factor 2B, subunit 3 gamma, 58kDa |
| 218494_s_at | Hs.435126 | SLC2A4RG | SLC2A4 regulator |
| 218527_at | Hs.20158 | APTX | aprataxin |
| 218533_s_at | Hs.504998 | UCKL1 | uridine-cytidine kise 1-like 1 |
| 218566_s_at | Hs.22857 | CHORDC1 | cysteine and histidine-rich domain (CHORD)-containing 1 |
| 218661_at | Hs.513296 | T15 | N-acetyltransferase 15 (GCN5-related, putative) |
| 218754_at | Hs.59425 | NOL9 | nucleolar protein 9 |
| 218982_s_at | Hs.44298 | MRPS17 /// ZNF713 | mitochondrial ribosomal protein S17 /// zinc finger protein 713 |
| 219065_s_at | Hs.444969 | DPY30 /// MEMO1 | dpy-30 homolog (C. elegans) /// mediator of cell motility 1 |
| 219120_at | Hs.24624 | C2orf44 | chromosome 2 open reading frame 44 |
| 219192_at | Hs.493739 | UBAP2 | ubiquitin associated protein 2 |
| 219679_s_at | Hs.435610 | WAC | WW domain containing adaptor with coiled-coil |
| 220606_s_at | Hs.47668 | C17orf48 | chromosome 17 open reading frame 48 |
| 220943_s_at | Hs.433466 | C2orf56 | chromosome 2 open reading frame 56 |
| 220947_s_at | Hs.632182 | TBC1D10B | TBC1 domain family, member 10B |
| 221230_s_at | Hs.575782 | ARID4B | AT rich interactive domain 4B (RBP1-like) |
| 221253_s_at | Hs.150837 | MUTED /// TXNDC5 | muted homolog (mouse) /// thioredoxin domain containing 5 (endoplasmic reticulum) |
| 221255_s_at | Hs.30011 | TMEM93 | transmembrane protein 93 |
| 221434_s_at | Hs.655105 | C14orf156 | chromosome 14 open reading frame 156 |
| 221488_s_at | Hs.520070 | CUTA | cutA divalent cation tolerance homolog (E. coli) |
| 221580_s_at | Hs.355750 | TAF1D | TATA box binding protein (TBP)-associated factor, R polymerase I, D, 41kDa |
| 221741_s_at | Hs.724397 | YTHDF1 | YTH domain family, member 1 |
| 221769_at | Hs.592080 | SPSB3 | splA/ryanodine receptor domain and SOCS box containing 3 |
| 221836_s_at | Hs.654911 | TRAPPC9 | trafficking protein particle complex 9 |
| 221923_s_at | Hs.557550 | NPM1 | nucleophosmin (nucleolar phosphoprotein B23, numatrin) |
| 222000_at | Hs.103939 | C1orf174 | chromosome 1 open reading frame 174 |
| 32029_at | Hs.459691 | PDPK1 | 3-phosphoinositide dependent protein kise-1 |
| 41512_at | Hs.530940 | BRAP | BRCA1 associated protein |
| 45526_g_at | Hs.513296 | T15 | N-acetyltransferase 15 (GCN5-related, putative) |
| 46256_at | Hs.592080 | SPSB3 | splA/ryanodine receptor domain and SOCS box containing 3 |
| 46270_at | Hs.268963 | UBAP1 | ubiquitin associated protein 1 |
| 56829_at | Hs.654911 | TRAPPC9 | trafficking protein particle complex 9 |
| 61874_at | Hs.62003 | C9orf7 | chromosome 9 open reading frame 7 |
