# Supplementary material for: A Systematic Evaluation of Multi-Gene Predictors for the Pathological Response of Breast Cancer Patients to Chemotherapy
Source: PLoS One. 2012 Nov 21;7(11):e49529. doi: 10.1371/journal.pone.0049529 (PMC3504014; doi:10.1371/journal.pone.0049529)
Supplement: Table S6 — MGP-TFAC developed from the Neve training set by the COXEN method. (DOC) [file pone.0049529.s006.doc]

Supplementary Table S6: MGP-TFAC developed from the Neve training sets by the COXEN method

| Probeset | UniGene.ID | Gene.Symbol | Gene.Title |
| --- | --- | --- | --- |
| 56829_at | Hs.116828/Hs.654911 | TRAPPC9 | trafficking protein particle complex 9 |
| 212977_at | Hs.471751 | CXCR7 | chemokine (C-X-C motif) receptor 7 |
| 207854_at | Hs.654368/Hs.728753 | GYPE | glycophorin E (MNS blood group) |
| 218390_s_at | Hs.372309 | C10orf84 | chromosome 10 open reading frame 84 |
| 221263_s_at | Hs.110695 | SF3B5 | splicing factor 3b, subunit 5, 10kDa |
| 218704_at | Hs.584916 | RNF43 | ring finger protein 43 |
| 200925_at | Hs.369624 | COX6A1 | cytochrome c oxidase subunit VIa polypeptide 1 |
| 212830_at | Hs.726061 | MEGF9 | multiple EGF-like-domains 9 |
| 218506_x_at | Hs.387255 | GLYR1 | glyoxylate reductase 1 homolog (Arabidopsis) |
| 200937_s_at | Hs.532359 | RPL5 | ribosomal protein L5 |
| 211519_s_at | Hs.720061 | KIF2C | kinesin family member 2C |
| 219653_at | Hs.105379 | LSM14B | LSM14B, SCD6 homolog B (S. cerevisiae) |
| 201010_s_at | Hs.533977 | TXNIP | thioredoxin interacting protein |
| 220295_x_at | Hs.445098 | DEPDC1 | DEP domain containing 1 |
| 202595_s_at | Hs.146585 | LEPROTL1 | leptin receptor overlapping transcript-like 1 |
| 200961_at | Hs.118725 | SEPHS2 | selenophosphate synthetase 2 |
| 218577_at | Hs.147836 | LRRC40 | leucine rich repeat containing 40 |
| 209180_at | Hs.78948 | RABGGTB | Rab geranylgeranyltransferase, beta subunit |
| 212700_x_at | Hs.514242 | PLEKHM1 | pleckstrin homology domain containing, family M (with RUN domain) member 1 |
| 202774_s_at | Hs.308171 | SFSWAP | splicing factor, suppressor of white-apricot homolog (Drosophila) |
| 202590_s_at | Hs.256667 | PDK2 | pyruvate dehydrogenase kinase, isozyme 2 |
| 203143_s_at | Hs.518138 | KIAA0040 | KIAA0040 |
| 212528_at | Hs.570455 | PPPDE2 | PPPDE peptidase domain containing 2 |
| 218226_s_at | Hs.304613 | NDUFB4 | NADH dehydrogenase (ubiquinone) 1 beta subcomplex, 4, 15kDa |
| 213798_s_at | Hs.370581/Hs.713078 | CAP1 | CAP, adenylate cyclase-associated protein 1 (yeast) |
| 219165_at | Hs.632034 | PDLIM2 | PDZ and LIM domain 2 (mystique) |
| 219395_at | Hs.436585/Hs.592053 | ESRP2 | epithelial splicing regulatory protein 2 |
| 218259_at | Hs.49143 | MKL2 | MKL/myocardin-like 2 |
| 209408_at | Hs.720061 | KIF2C | kinesin family member 2C |
| 212845_at | Hs.98259 | SAMD4A | sterile alpha motif domain containing 4A |
| 209191_at | Hs.193491/Hs.725915 | TUBB6 | tubulin, beta 6 |
| 211954_s_at | Hs.712598 | IPO5 | importin 5 |
| 212465_at | Hs.510407 | SETD3 | SET domain containing 3 |
| 207986_x_at | Hs.355264 | CYB561 | cytochrome b-561 |
| 209221_s_at | Hs.473254 | OSBPL2 | oxysterol binding protein-like 2 |
| 218640_s_at | Hs.29724 | PLEKHF2 | pleckstrin homology domain containing, family F (with FYVE domain) member 2 |
| 209459_s_at | Hs.336768 | ABAT | 4-aminobutyrate aminotransferase |
| 221580_s_at | Hs.355750 | TAF1D | TATA box binding protein (TBP)-associated factor, RNA polymerase I, D, 41kDa |
| 222234_s_at | Hs.301394 | DBNDD1 | dysbindin (dystrobrevin binding protein 1) domain containing 1 |
| 218494_s_at | Hs.435126 | SLC2A4RG | SLC2A4 regulator |
| 213237_at | Hs.585209 | C16orf88 | chromosome 16 open reading frame 88 |
| 203707_at | Hs.611475 | ZNF263 | zinc finger protein 263 |
| 212367_at | Hs.362733 | FEM1B | fem-1 homolog b (C. elegans) |
| 205413_at | Hs.289795 | MPPED2 | metallophosphoesterase domain containing 2 |
| 209669_s_at | Hs.530412 | SERBP1 | SERPINE1 mRNA binding protein 1 |
| 204352_at | Hs.523930 | TRAF5 | TNF receptor-associated factor 5 |
| 209460_at | Hs.336768 | ABAT | 4-aminobutyrate aminotransferase |
