# Supplementary material for: A Systematic Evaluation of Multi-Gene Predictors for the Pathological Response of Breast Cancer Patients to Chemotherapy
Source: PLoS One. 2012 Nov 21;7(11):e49529. doi: 10.1371/journal.pone.0049529 (PMC3504014; doi:10.1371/journal.pone.0049529)
Supplement: Table S7 — MGP-FEC developed from the Neve training set by the COXEN method. (DOC) [file pone.0049529.s007.doc]

Supplementary Table S7: MGP-FEC developed from the Neve training sets by the COXEN method.

| Probeset | UniGene.ID | Gene.Symbol | Gene.Title |
| --- | --- | --- | --- |
| 211954_s_at | Hs.712598 | IPO5 | importin 5 |
| 211955_at | Hs.712598 | IPO5 | importin 5 |
| 219395_at | Hs.592053 | ESRP2 | epithelial splicing regulatory protein 2 |
| 206506_s_at | Hs.368325 | SUPT3H | suppressor of Ty 3 homolog (S. cerevisiae) |
| 217604_at |  |  |  |
| 216463_at | Hs.541694 | AL117520 |  |
| 205961_s_at | Hs.726445 | PSIP1 | PC4 and SFRS1 interacting protein 1 |
| 209337_at | Hs.726445 | PSIP1 | PC4 and SFRS1 interacting protein 1 |
| 209209_s_at | Hs.509343 | FERMT2 | fermitin family member 2 |
| 209610_s_at | Hs.654352 | SLC1A4 | solute carrier family 1 (glutamate/neutral amino acid transporter), member 4 |
| 201426_s_at | Hs.455493 | VIM | vimentin |
| 219532_at | Hs.101915 | ELOVL4 | elongation of very long chain fatty acids (FEN1/Elo2, SUR4/Elo3, yeast)-like 4 |
| 209736_at | Hs.201671 | SOX13 | SRY (sex determining region Y)-box 13 |
| 204458_at | Hs.632199 | PLA2G15 | phospholipase A2, group XV |
| 1729_at | Hs.460996 | TRADD | TNFRSF1A-associated via death domain |
| 200966_x_at | Hs.513490 | ALDOA | aldolase A, fructose-bisphosphate |
| 202521_at | Hs.368367 | CTCF | CCCTC-binding factor (zinc finger protein) |
| 218506_x_at | Hs.387255 | GLYR1 | glyoxylate reductase 1 homolog (Arabidopsis) |
| 212700_x_at | Hs.514242 | PLEKHM1 | pleckstrin homology domain containing, family G (with RhoGef domain) member 1 |
| 201131_s_at | Hs.461086 | CDH1 | cadherin 1, type 1, E-cadherin (epithelial) |
| 214687_x_at | Hs.513490 | ALDOA | aldolase A, fructose-bisphosphate |
| 209512_at | Hs.59486 | HSDL2 | hydroxysteroid dehydrogenase like 2 |
| 221263_s_at | Hs.110695 | SF3B5 | splicing factor 3b, subunit 5, 10kDa |
| 213573_at | Hs.595245 | KPNB1 | karyopherin (importin) beta 1 |
| 212322_at | Hs.499984 | SGPL1 | sphingosine-1-phosphate lyase 1 |
| 206302_s_at | Hs.601274 | NUDT4 | nudix (nucleoside diphosphate linked moiety X)-type motif 4 |
| 215123_at | Hs.661773 |  |  |
| 214024_s_at | Hs.410965 | DGCR6L | DiGeorge syndrome critical region gene 6-like |
| 218904_s_at | Hs.532296 | C9orf40 | chromosome 9 open reading frame 40 |
| 43544_at | Hs.365207 | MED16 | mediator complex subunit 16 |
| 202595_s_at | Hs.146585 | LEPROTL1 | leptin receptor overlapping transcript-like 1 |
| 201853_s_at | Hs.153752 | CDC25B | cell division cycle 25 homolog B (S. pombe) |
| 215696_s_at | Hs.522500/Hs.668588 | SEC16A | SEC16 homolog A (S. cerevisiae) |
| 221809_at | Hs.368569 | RANBP10 | RAN binding protein 10 |
| 221545_x_at | Hs.365207 | MED16 | mediator complex subunit 16 |
| 212617_at | Hs.714372 | ZNF609 | zinc finger protein 609 |
| 204909_at | Hs.408461 | DDX6 | DEAD (Asp-Glu-Ala-Asp) box polypeptide 6 |
| 202003_s_at | Hs.200136 | ACAA2 | acetyl-CoA acyltransferase 2 |
| 210058_at | Hs.178695 | MAPK13 | mitogen-activated protein kinase 13 |
| 209101_at | Hs.410037 | CTGF | connective tissue growth factor |
| 213035_at | Hs.335239 | ANKRD28 | ankyrin repeat domain 28 |
| 203692_s_at | Hs.269408 | E2F3 | E2F transcription factor 3 |
| 212810_s_at | Hs.654352 | SLC1A4 | solute carrier family 1 (glutamate/neutral amino acid transporter), member 4 |
| 205641_s_at | Hs.460996 | TRADD | TNFRSF1A-associated via death domain |
| 208146_s_at | Hs.233389 | CPVL | carboxypeptidase, vitellogenic-like |
| 209884_s_at | Hs.250072 | SLC4A7 | solute carrier family 4, sodium bicarbonate cotransporter, member 7 |
| 200087_s_at | Hs.75914 | TMED2 | transmembrane emp24 domain trafficking protein 2 |
| 202064_s_at | Hs.181300 | SEL1L | sel-1 suppressor of lin-12-like (C. elegans) |
| 200044_at | Hs.706889 | SRSF9 | serine/arginine-rich splicing factor 9 |
| 212181_s_at | Hs.601274 | NUDT4 | nudix (nucleoside diphosphate linked moiety X)-type motif 4 |
| 221566_s_at | Hs.513667 | NOL3 | nucleolar protein 3 (apoptosis repressor with CARD domain) |
| 200937_s_at | Hs.532359 | RPL5 | ribosomal protein L5 |
| 219924_s_at | Hs.675613 | ZMYM6 | zinc finger, MYM-type 6 |
| 208936_x_at | Hs.4082 | LGALS8 | lectin, galactoside-binding, soluble, 8 |
| 59625_at | Hs.513667 | NOL3 | nucleolar protein 3 (apoptosis repressor with CARD domain) |
| 203939_at | Hs.153952 | NT5E | 5'-nucleotidase, ecto (CD73) |
| 213574_s_at | Hs.595245 | KPNB1 | karyopherin (importin) beta 1 |
| 38918_at | Hs.201671 | SOX13 | SRY (sex determining region Y)-box 13 |
| 218959_at | Hs.44276 | HOXC10 | homeobox C10 |
| 213223_at | Hs.652114 | RPL28 | ribosomal protein L28 |
| 221418_s_at | Hs.365207 | MED16 | mediator complex subunit 16 |
| 210286_s_at | Hs.250072 | SLC4A7 | solute carrier family 4, sodium bicarbonate cotransporter, member 7 |
| 217844_at | Hs.444468 | CTDSP1 | CTD (carboxy-terminal domain, RNA polymerase II, polypeptide A) small phosphatase 1 |
| 209513_s_at | Hs.59486 | HSDL2 | hydroxysteroid dehydrogenase like 2 |
| 43977_at | Hs.631629 | TMEM161A | transmembrane protein 161A |
| 217018_at |  |  |  |
| 219088_s_at | Hs.11110 | ZNF576 | zinc finger protein 576 |
| 213080_x_at | Hs.180946 | RPL5 | ribosomal protein L5 |
| 218301_at | Hs.5345 | RNPEPL1 | arginyl aminopeptidase (aminopeptidase B)-like 1 |
| 211953_s_at | Hs.712598 | IPO5 | importin 5 |
| 213303_x_at | Hs.591384 | ZBTB7A | zinc finger and BTB domain containing 7A |
| 53987_at | Hs.368569 | RANBP10 | RAN binding protein 10 |
| 218661_at | Hs.513296 | NAT15 | N-acetyltransferase 15 (GCN5-related, putative) |
| 210965_x_at | Hs.233552 | CDK13 | cyclin-dependent kinase 13 |
| 37547_at | Hs.372360 | BBS9 | Bardet-Biedl syndrome 9 |
| 210059_s_at | Hs.178695 | MAPK13 | mitogen-activated protein kinase 13 |
| 221567_at | Hs.513667 | NOL3 | nucleolar protein 3 (apoptosis repressor with CARD domain) |
| 212692_s_at | Hs.480938 | LRBA | LPS-responsive vesicle trafficking, beach and anchor containing |
| 204140_at | Hs.421194 | TPST1 | tyrosylprotein sulfotransferase 1 |
| 74694_s_at | Hs.555978 | RABEP2 | rabaptin, RAB GTPase binding effector protein 2 |
| 214557_at | Hs.668806 | PTTG2 | pituitary tumor-transforming 2 |
| 218466_at | Hs.631587 | TBC1D17 | TBC1 domain family, member 17 |
| 213258_at | Hs.516578 | TFPI | tissue factor pathway inhibitor (lipoprotein-associated coagulation inhibitor) |
| 218488_at | Hs.533549 | EIF2B3 | eukaryotic translation initiation factor 2B, subunit 3 gamma, 58kDa |
| 217795_s_at | Hs.517817 | TMEM43 | transmembrane protein 43 |
| 208933_s_at | Hs.4082/Hs.708114 | LGALS8 | lectin, galactoside-binding, soluble, 8 |
| 203707_at | Hs.611475 | ZNF263 | zinc finger protein 263 |
| 205316_at | Hs.518089 | SLC15A2 | solute carrier family 15 (H+/peptide transporter), member 2 |
| 205981_s_at | Hs.107153 | ING2 | inhibitor of growth family, member 2 |
| 218227_at | Hs.256549 | NUBP2 | nucleotide binding protein 2 (MinD homolog, E. coli) |
| 212823_s_at | Hs.509637 | PLEKHG3 | pleckstrin homology domain containing, family G (with RhoGef domain) member 3 |
| 211452_x_at | Hs.471779 | LRRFIP1 | leucine rich repeat (in FLII) interacting protein 1 |
| 213803_at | Hs.532793/Hs.706168 | KPNB1 | karyopherin (importin) beta 1 |
| 46256_at | Hs.592080 | SPSB3 | splA/ryanodine receptor domain and SOCS box containing 3 |
| 201650_at | Hs.654568 | KRT19 | keratin 19 |
| 214843_s_at | Hs.726151 | USP33 | ubiquitin specific peptidase 33 |
| 218010_x_at | Hs.79625 | PPDPF | pancreatic progenitor cell differentiation and proliferation factor homolog (zebrafish) |
| 221767_x_at | Hs.471851 | HDLBP | high density lipoprotein binding protein |
| 212845_at | Hs.98259 | SAMD4A | sterile alpha motif domain containing 4A |
| 213141_at | Hs.513683 | PSKH1 | protein serine kinase H1 |
| 205100_at | Hs.696497 | GFPT2 | glutamine-fructose-6-phosphate transaminase 2 |
| 207604_s_at | Hs.250072 | SLC4A7 | solute carrier family 4, sodium bicarbonate cotransporter, member 7 |
| 221696_s_at | Hs.24979 | STYK1 | serine/threonine/tyrosine kinase 1 |
| 212193_s_at | Hs.292078 | LARP1 | La ribonucleoprotein domain family, member 1 |
| 202194_at | Hs.482873 | TMED5 | transmembrane emp24 protein transport domain containing 5 |
| 204679_at | Hs.208544 | KCNK1 | potassium channel, subfamily K, member 1 |
| 204392_at | Hs.434875 | CAMK1 | calcium/calmodulin-dependent protein kinase I |
| 202738_s_at | Hs.78060 | PHKB | phosphorylase kinase, beta |
| 203369_x_at | Hs.533040 | PDLIM7 | PDZ and LIM domain 7 (enigma) |
| 206043_s_at | Hs.6168 | ATP2C2 | ATPase, Ca++ transporting, type 2C, member 2 |
| 219382_at | Hs.515412 | SERTAD3 | SERTA domain containing 3 |
| 202774_s_at | Hs.308171 | SFSWAP | splicing factor, suppressor of white-apricot homolog (Drosophila) |
| 218683_at | Hs.596061/Hs.726436 | PTBP2 | polypyrimidine tract binding protein 2 |
| 202739_s_at | Hs.78060 | PHKB | phosphorylase kinase, beta |
| 218494_s_at | Hs.435126 | SLC2A4RG | SLC2A4 regulator |
| 213429_at | Hs.100261 |  |  |
| 217979_at | Hs.364544 | TSPAN13 | tetraspanin 13 |
| 202686_s_at | Hs.590970 | AXL | AXL receptor tyrosine kinase |
| 203196_at | Hs.508423 | ABCC4 | ATP-binding cassette, sub-family C (CFTR/MRP), member 4 |
| 205802_at | Hs.250687 | TRPC1 | transient receptor potential cation channel, subfamily C, member 1 |
| 212792_at | Hs.408623 | DPY19L1 | dpy-19-like 1 (C. elegans) |
| 218245_at | Hs.8361 | TSKU | tsukushi small leucine rich proteoglycan homolog (Xenopus laevis) |
| 212811_x_at | Hs.654352 | SLC1A4 | solute carrier family 1 (glutamate/neutral amino acid transporter), member 4 |
| 201863_at | Hs.631614 | FAM32A | family with sequence similarity 32, member A |
| 213698_at | Hs.533986/Hs.623978/Hs.729053 | ZMYM6 | zinc finger, MYM-type 6 |
| 213743_at | Hs.658324 | CCNT2 | cyclin T2 |
| 204540_at | Hs.433839 | EEF1A2 | eukaryotic translation elongation factor 1 alpha 2 |
| 204011_at | Hs.18676 | SPRY2 | sprouty homolog 2 (Drosophila) |
| 209210_s_at | Hs.509343 | FERMT2 | fermitin family member 2 |
| 202888_s_at | Hs.1239 | ANPEP | alanyl (membrane) aminopeptidase |
| 210014_x_at | Hs.436405 | IDH3B | isocitrate dehydrogenase 3 (NAD+) beta |
| 201698_s_at | Hs.706889/Hs.728777 | SRSF9 | serine/arginine-rich splicing factor 9 |
| 202760_s_at | Hs.591908 | PALM2-AKAP2. | PALM2-AKAP2 readthrough |
| 208308_s_at | Hs.466471 | GPI | glucose-6-phosphate isomerase |
| 205120_s_at | Hs.438953 | SGCB | sarcoglycan, beta (43kDa dystrophin-associated glycoprotein) |
| 201315_x_at | Hs.709321 | IFITM2 | interferon induced transmembrane protein 2 (1-8D) |
| 206953_s_at | Hs.24212/Hs.649282 | LPHN2 | latrophilin 2 |
| 205996_s_at | Hs.470907 | AK2 | adenylate kinase 2 |
| 204646_at | Hs.335034 | DPYD | dihydropyrimidine dehydrogenase |
| 219653_at | Hs.105379 | LSM14B | LSM14B, SCD6 homolog B (S. cerevisiae) |
| 210825_s_at | Hs.433863 | PEBP1 | phosphatidylethanolamine binding protein 1 |
| 204115_at | Hs.83381 | GNG11 | guanine nucleotide binding protein (G protein), gamma 11 |
| 204556_s_at | Hs.656580 | DZIP1 | DAZ interacting protein 1 |
| 206303_s_at | Hs.506325 | NUDT4 | nudix (nucleoside diphosphate linked moiety X)-type motif 4 |
| 222193_at | Hs.187823 | C2orf43 | chromosome 2 open reading frame 43 |
| 210186_s_at | Hs.471933/Hs.700839 | FKBP1A | FK506 binding protein 1A, 12kDa |
| 208934_s_at | Hs.4082/Hs.708114 | LGALS8 | lectin, galactoside-binding, soluble, 8 |
| 208921_s_at | Hs.489040 | SRI | sorcin |
| 210673_x_at | Hs.94367 | NKX2-1 | NK2 homeobox 1 |
| 218035_s_at | Hs.518727 | RBM47 | RNA binding motif protein 47 |
| 211952_at | Hs.712598 | IPO5 | importin 5 |
| 217939_s_at | Hs.655167 | AFTPH | aftiphilin |
| 204588_s_at | Hs.513147 | SLC7A7 | solute carrier family 7 (cationic amino acid transporter, y+ system), member 7 |
| 211316_x_at | Hs.390736 | CFLAR | CASP8 and FADD-like apoptosis regulator |
| 220318_at | Hs.670090 | EPN3 | epsin 3 |
| 216565_x_at |  |  |  |
| 218390_s_at | Hs.372309 | C10orf84 | chromosome 10 open reading frame 84 |
| 201514_s_at | Hs.587054 | G3BP1 | GTPase activating protein (SH3 domain) binding protein 1 |
| 203650_at | Hs.647450 | PROCR | protein C receptor, endothelial |
| 200851_s_at | Hs.232194 | KIAA0174 | KIAA0174 |
| 218577_at | Hs.147836 | LRRC40 | leucine rich repeat containing 40 |
| 201292_at | Hs.156346 | TOP2A | topoisomerase (DNA) II alpha 170kDa |
