# Supplementary material for: A Systematic Evaluation of Multi-Gene Predictors for the Pathological Response of Breast Cancer Patients to Chemotherapy
Source: PLoS One. 2012 Nov 21;7(11):e49529. doi: 10.1371/journal.pone.0049529 (PMC3504014; doi:10.1371/journal.pone.0049529)
Supplement: Table S8 — MGP-TFAC developed from the Hoeflich training set by the COXEN method. (DOC) [file pone.0049529.s008.doc]

Supplementary Table S8: MGP-TFAC developed from the Hoeflich training sets by the COXEN method.

| Probeset | UniGene.ID | Gene.Symbol | Gene.Title |
| --- | --- | --- | --- |
| 205594_at | Hs.463375 | ZNF652 | zinc finger protein 652 |
| 217294_s_at | Hs.517145 | ENO1 | enolase 1, (alpha) |
| 56829_at | Hs.654911 | TRAPPC9 | trafficking protein particle complex 9 |
| 215696_s_at | Hs.705608 | SEC16A | SEC16 homolog A (S. cerevisiae) |
| 220606_s_at | Hs.47668 | C17orf48 | chromosome 17 open reading frame 48 |
| 41512_at | Hs.530940 | BRAP | BRCA1 associated protein |
| 212046_x_at | Hs.861 | MAPK3 | mitogen-activated protein kinase 3 |
| 215707_s_at | Hs.472010 | PRNP | prion protein |
| 211958_at | Hs.607212 | IGFBP5 | insulin-like growth factor binding protein 5 |
| 205479_s_at | Hs.77274 | PLAU | plasminogen activator, urokinase |
| 206972_s_at | Hs.271809 | GPR161 | G protein-coupled receptor 161 |
| 208935_s_at | Hs.4082 | LGALS8 | lectin, galactoside-binding, soluble, 8 |
| 216204_at | Hs.713616 | ARVCF | armadillo repeat gene deleted in velocardiofacial syndrome |
| 203424_s_at | Hs.607212 | IGFBP5 | insulin-like growth factor binding protein 5 |
| 219127_at | Hs.368260 | PRR15L | proline rich 15-like |
| 208936_x_at | Hs.4082 | LGALS8 | lectin, galactoside-binding, soluble, 8 |
| 218566_s_at | Hs.22857 | CHORDC1 | cysteine and histidine-rich domain (CHORD) containing 1 |
| 212508_at | Hs.24719 | MOAP1 | modulator of apoptosis 1 |
| 209494_s_at | Hs.728132 | PATZ1 | POZ (BTB) and AT hook containing zinc finger 1 |
| 203425_s_at | Hs.607212 | IGFBP5 | insulin-like growth factor binding protein 5 |
| 202704_at | Hs.714780 | TOB1 | transducer of ERBB2, 1 |
| 212692_s_at | Hs.480938 | LRBA | LPS-responsive vesicle trafficking, beach and anchor containing |
| 202106_at | Hs.507333 | GOLGA3 | golgin A3 |
| 202076_at | Hs.696238 | BIRC2 | baculoviral IAP repeat containing 2 |
| 213196_at | Hs.301094 | ZNF629 | zinc finger protein 629 |
| 212367_at | Hs.362733 | FEM1B | fem-1 homolog b (C. elegans) |
| 211668_s_at | Hs.77274 | PLAU | plasminogen activator, urokinase |
| 208933_s_at | Hs.4082 | LGALS8 | lectin, galactoside-binding, soluble, 8 |
| 211421_s_at | Hs.350321 | RET | ret proto-oncogene |
| 201231_s_at | Hs.517145 | ENO1 | enolase 1, (alpha) |
| 201323_at | Hs.346868 | EBNA1BP2 | EBNA1 binding protein 2 |
| 209087_x_at | Hs.599039 | MCAM | melanoma cell adhesion molecule |
| 215552_s_at | Hs.208124 | ESR1 | estrogen receptor 1 |
| 214104_at | Hs.271809 | GPR161 | G protein-coupled receptor 161 |
| 202204_s_at | Hs.295137 | AMFR | autocrine motility factor receptor |
| 204951_at | Hs.654594 | RHOH | ras homolog gene family, member H |
| 218770_s_at | Hs.25544 | TMEM39B | transmembrane protein 39B |
| 221003_s_at | Hs.87159 | CAB39L | calcium binding protein 39-like |
| 202636_at | Hs.725984 | RNF103 | ring finger protein 103 |
| 213781_at | Hs.285363 | LRRC68 | leucine rich repeat containing 68 |
| 209460_at | Hs.336768 | ABAT | 4-aminobutyrate aminotransferase |
| 201176_s_at | Hs.33642 | ARCN1 | archain 1 |
| 202743_at | Hs.655387 | PIK3R3 | phosphoinositide-3-kinase, regulatory subunit 3 (gamma) |
| 213743_at | Hs.658324 | CCNT2 | cyclin T2 |
| 203821_at | Hs.799 | HBEGF | heparin-binding EGF-like growth factor |
| 211959_at | Hs.607212 | IGFBP5 | insulin-like growth factor binding protein 5 |
| 202998_s_at | Hs.626637 | LOXL2 | lysyl oxidase-like 2 |
| 218597_s_at | Hs.370102 | CISD1 | CDGSH iron sulfur domain 1 |
| 212400_at | Hs.535972 | FAM102A | family with sequence similarity 102, member A |
| 209101_at | Hs.410037 | CTGF | connective tissue growth factor |
| 217939_s_at | Hs.655167 | AFTPH | aftiphilin |
| 209623_at | Hs.604789 | MCCC2 | methylcrotonoyl-CoA carboxylase 2 (beta) |
| 221869_at | Hs.729072 | ZNF512B | zinc finger protein 512B |
| 208921_s_at | Hs.489040 | SRI | sorcin |
| 202760_s_at | Hs.591908 | PALM2-AKAP2 | PALM2-AKAP2 readthrough |
| 62987_r_at | Hs.514423 | CACNG4 | calcium channel, voltage-dependent, gamma subunit 4 |
| 204969_s_at | Hs.263671 | RDX | radixin |
| 212246_at | Hs.293689 | MCFD2 | multiple coagulation factor deficiency 2 |
| 203426_s_at | Hs.607212 | IGFBP5 | insulin-like growth factor binding protein 5 |
| 207998_s_at | Hs.476358 | CACNA1D | calcium channel, voltage-dependent, L type, alpha 1D subunit |
| 204194_at | Hs.154276 | BACH1 | BTB and CNC homology 1, basic leucine zipper transcription factor 1 |
| 46256_at | Hs.592080 | SPSB3 | splA/ryanodine receptor domain and SOCS box containing 3 |
| 206923_at | Hs.531704 | PRKCA | protein kinase C, alpha |
| 208741_at | Hs.524899 | SAP18 | Sin3A-associated protein, 18kDa |
| 207334_s_at | Hs.82028 | TGFBR2 | transforming growth factor, beta receptor II (70/80kDa) |
| 202452_at | Hs.147950 | ZER1 | zer-1 homolog (C. elegans) |
| 209209_s_at | Hs.509343 | FERMT2 | fermitin family member 2 |
| 210005_at | Hs.473648 | GART | phosphoribosylglycinamide formyltransferase, phosphoribosylglycinamide synthetase, phosphoribosylaminoimidazole synthetase |
| 203754_s_at | Hs.424484 | BRF1 | BRF1 homolog, subunit of RNA polymerase III transcription initiation factor IIIB (S. cerevisiae) |
| 202997_s_at | Hs.626637 | LOXL2 | lysyl oxidase-like 2 |
| 212441_at | Hs.79276 | KIAA0232 | KIAA0232 |
| 204268_at | Hs.516484 | S100A2 | S100 calcium binding protein A2 |
| 204977_at | Hs.591931 | DDX10 | DEAD (Asp-Glu-Ala-Asp) box polypeptide 10 |
| 203614_at | Hs.512963 | UTP14C | UTP14, U3 small nucleolar ribonucleoprotein, homolog C (yeast) |
| 208763_s_at | Hs.728167 | TSC22D3 | TSC22 domain family, member 3 |
| 203065_s_at | Hs.74034 | CAV1 | caveolin 1, caveolae protein, 22kDa |
| 221580_s_at | Hs.355750 | TAF1D | TATA box binding protein (TBP)-associated factor, RNA polymerase I, D, 41kDa |
| 61874_at | Hs.62003 | C9orf7 | chromosome 9 open reading frame 7 |
| 215543_s_at | Hs.474667 | LARGE | like-glycosyltransferase |
| 216218_s_at | Hs.727618 | PLCL2 | phospholipase C-like 2 |
| 201329_s_at | Hs.644231 | ETS2 | v-ets erythroblastosis virus E26 oncogene homolog 2 (avian) |
| 219223_at | Hs.62003 | C9orf7 | chromosome 9 open reading frame 7 |
| 203870_at | Hs.7966 | USP46 | ubiquitin specific peptidase 46 |
| 205862_at | Hs.467733 | GREB1 | growth regulation by estrogen in breast cancer 1 |
| 205151_s_at | Hs.21572 | TRIL | TLR4 interactor with leucine-rich repeats |
| 222303_at | NA | NA | NA |
| 219041_s_at | Hs.647086 | REPIN1 | replication initiator 1 |
| 218379_at | Hs.7527 | RBM7 | RNA binding motif protein 7 |
| 204667_at | Hs.163484 | FOXA1 | forkhead box A1 |
| 202643_s_at | Hs.211600 | TNFAIP3 | tumor necrosis factor, alpha-induced protein 3 |
| 211340_s_at | Hs.599039 | MCAM | melanoma cell adhesion molecule |
| 205225_at | Hs.208124 | ESR1 | estrogen receptor 1 |
| 206653_at | Hs.282387 | POLR3G | polymerase (RNA) III (DNA directed) polypeptide G (32kD) |
| 212593_s_at | Hs.711490 | PDCD4 | programmed cell death 4 (neoplastic transformation inhibitor) |
| 208934_s_at | Hs.4082/Hs.708114 | LGALS8 | lectin, galactoside-binding, soluble, 8 |
| 209459_s_at | Hs.336768 | ABAT | 4-aminobutyrate aminotransferase |
| 214109_at | Hs.480938 | LRBA | LPS-responsive vesicle trafficking, beach and anchor containing |
| 201276_at | Hs.567328 | RAB5B | RAB5B, member RAS oncogene family |
| 210652_s_at | Hs.112949 | TTC39A | tetratricopeptide repeat domain 39A |
| 220277_at | Hs.12248 | CXXC4 | CXXC finger protein 4 |
| 200054_at | Hs.7165 | ZNF259 | zinc finger protein 259 |
| 200711_s_at | Hs.171626 | SKP1 | S-phase kinase-associated protein 1 |
| 217445_s_at | Hs.473648 | GART | phosphoribosylglycinamide formyltransferase, phosphoribosylglycinamide synthetase, phosphoribosylaminoimidazole synthetase |
| 213508_at | Hs.269909 | C14orf147 | chromosome 14 open reading frame 147 |
| 210869_s_at | Hs.599039 | MCAM | melanoma cell adhesion molecule |
| 205996_s_at | Hs.470907 | AK2 | adenylate kinase 2 |
| 202644_s_at | Hs.211600 | TNFAIP3 | tumor necrosis factor, alpha-induced protein 3 |
| 208637_x_at | Hs.509765 | ACTN1 | actinin, alpha 1 |
| 208736_at | Hs.524741 | ARPC3 | actin related protein 2/3 complex, subunit 3, 21kDa |
| 204199_at | Hs.648175 | RALGPS1 | Ral GEF with PH domain and SH3 binding motif 1 |
| 202718_at | Hs.438102 | IGFBP2 | insulin-like growth factor binding protein 2, 36kDa |
| 204420_at | Hs.283565 | FOSL1 | FOS-like antigen 1 |
| 209196_at | Hs.520063 | WDR46 | WD repeat domain 46 |
| 219956_at | Hs.505575 | GALNT6 | UDP-N-acetyl-alpha-D-galactosamine:polypeptide N-acetylgalactosaminyltransferase 6 (GalNAc-T6) |
| 202558_s_at | Hs.352341 | HSPA13 | heat shock protein 70kDa family, member 13 |
| 212099_at | Hs.502876 | RHOB | ras homolog gene family, member B |
| 210108_at | Hs.476358 | CACNA1D | calcium channel, voltage-dependent, L type, alpha 1D subunit |
| 202708_s_at | Hs.2178 | HIST2H2BE | histone cluster 2, H2be |
| 203154_s_at | Hs.20447 | PAK4 | p21 protein (Cdc42/Rac)-activated kinase 4 |
| 218307_at | Hs.8033 | RSAD1 | radical S-adenosyl methionine domain containing 1 |
| 204287_at | Hs.216226 | SYNGR1 | synaptogyrin 1 |
| 218086_at | Hs.719906 | NPDC1 | neural proliferation, differentiation and control, 1 |
| 45526_g_at | Hs.513296 | NAT15 | N-acetyltransferase 15 (GCN5-related, putative) |
| 203405_at | Hs.473838 | PSMG1 | proteasome (prosome, macropain) assembly chaperone 1 |
