# Supplementary material for: A Systematic Evaluation of Multi-Gene Predictors for the Pathological Response of Breast Cancer Patients to Chemotherapy
Source: PLoS One. 2012 Nov 21;7(11):e49529. doi: 10.1371/journal.pone.0049529 (PMC3504014; doi:10.1371/journal.pone.0049529)
Supplement: Table S9 — MGP-FEC developed from the Hoeflich training set by the COXEN method. (DOC) [file pone.0049529.s009.doc]

Supplementary Table S9: MGP-FEC developed from the Hoeflich training sets by the COXEN method.

| Probeset | UniGene.ID | Gene.Symbol | Gene.Title |
| --- | --- | --- | --- |
| 56829_at | Hs.654911 | TRAPPC9 | trafficking protein particle complex 9 |
| 211421_s_at | Hs.350321 | RET | ret proto-oncogene |
| 215707_s_at | Hs.472010 | PRNP | prion protein |
| 205479_s_at | Hs.77274 | PLAU | plasminogen activator, urokinase |
| 215696_s_at | Hs.705608 | SEC16A | SEC16 homolog A (S. cerevisiae) |
| 217294_s_at | Hs.517145 | ENO1 | enolase 1, (alpha) |
| 218566_s_at | Hs.22857 | CHORDC1 | cysteine and histidine-rich domain (CHORD) containing 1 |
| 211668_s_at | Hs.77274 | PLAU | plasminogen activator, urokinase |
| 205594_at | Hs.463375 | ZNF652 | zinc finger protein 652 |
| 206972_s_at | Hs.271809 | GPR161 | G protein-coupled receptor 161 |
| 220606_s_at | Hs.47668 | C17orf48 | chromosome 17 open reading frame 48 |
| 209101_at | Hs.410037 | CTGF | connective tissue growth factor |
| 203754_s_at | Hs.424484 | BRF1 | BRF1 homolog, subunit of RNA polymerase III transcription initiation factor IIIB (S. cerevisiae) |
| 212046_x_at | Hs.861 | MAPK3 | mitogen-activated protein kinase 3 |
| 212367_at | Hs.362733 | FEM1B | fem-1 homolog b (C. elegans) |
| 219164_s_at | Hs.168241 | ATG2B | ATG2 autophagy related 2 homolog B (S. cerevisiae) |
| 215552_s_at | Hs.208124 | ESR1 | estrogen receptor 1 |
| 202743_at | Hs.655387 | PIK3R3 | phosphoinositide-3-kinase, regulatory subunit 3 (gamma) |
| 200835_s_at | Hs.517949 | MAP4 | microtubule-associated protein 4 |
| 209460_at | Hs.336768 | ABAT | 4-aminobutyrate aminotransferase |
