# Supplementary material for: A Systematic Evaluation of Multi-Gene Predictors for the Pathological Response of Breast Cancer Patients to Chemotherapy
Source: PLoS One. 2012 Nov 21;7(11):e49529. doi: 10.1371/journal.pone.0049529 (PMC3504014; doi:10.1371/journal.pone.0049529)
Supplement: Table S10 — MGP-TFAC developed from the ER positive Neve training set by the COXEN method. (DOC) [file pone.0049529.s010.doc]

Supplementary Table S10: MGP-TFAC developed from the ER positive Neve training sets by the COXEN method.

| Probeset | UniGene.ID | Gene.Symbol | Gene.Title |
| --- | --- | --- | --- |
| 204291_at | Hs.600823 | ZNF518A | zinc finger protein 518A |
| 204798_at | Hs.606320 | MYB | v-myb myeloblastosis viral oncogene homolog (avian) |
| 208003_s_at | Hs.371987 | NFAT5 | nuclear factor of activated T-cells 5, tonicity-responsive |
| 212231_at | Hs.728146 | FBXO21 | F-box protein 21 |
| 215923_s_at | Hs.516306 | PSD4 | pleckstrin and Sec7 domain containing 4 |
| 213025_at | Hs.460232 | THUMPD1 | THUMP domain containing 1 |
