# Supplementary material for: A Systematic Evaluation of Multi-Gene Predictors for the Pathological Response of Breast Cancer Patients to Chemotherapy
Source: PLoS One. 2012 Nov 21;7(11):e49529. doi: 10.1371/journal.pone.0049529 (PMC3504014; doi:10.1371/journal.pone.0049529)
Supplement: Table S11 — MGP-TFAC developed from the ER negative Neve training set by the COXEN method. (DOC) [file pone.0049529.s011.doc]

Supplementary Table S11: MGP-TFAC developed from the ER negative Neve training sets by the COXEN method.

| Probeset | UniGene.ID | Gene.Symbol | Gene.Title |
| --- | --- | --- | --- |
| 209380_s_at | Hs.368563 | ABCC5 | ATP-binding cassette, sub-family C (CFTR/MRP), member 5 |
| 218619_s_at | Hs.522639 | SUV39H1 | suppressor of variegation 3-9 homolog 1 (Drosophila) |
| 205517_at | Hs.243987 | GATA4 | GATA binding protein 4 |
| 208377_s_at | Hs.632799 | CACNA1F | calcium channel, voltage-dependent, L type, alpha 1F subunit |
| 205774_at | Hs.1321 | F12 | coagulation factor XII (Hageman factor) |
| 206024_at | Hs.2899 | HPD | 4-hydroxyphenylpyruvate dioxygenase |
| 211256_x_at | Hs.159028 | BTN2A1 | butyrophilin, subfamily 2, member A1 |
| 214154_s_at | Hs.164384 | PKP2 | plakophilin 2 |
| 41037_at | Hs.94865 | TEAD4 | TEA domain family member 4 |
| 31846_at | Hs.15114 | RHOD | ras homolog gene family, member D |
| 214157_at | Hs.125898 | GNAS | GNAS complex locus |
| 55872_at | Hs.729072 | ZNF512B | zinc finger protein 512B |
| 220318_at | Hs.670090 | EPN3 | epsin 3 |
| 207679_at | Hs.42146 | PAX3 | paired box 3 |
| 215493_x_at | Hs.159028 | BTN2A1 | butyrophilin, subfamily 2, member A1 |
| 219018_s_at | Hs.709288 | CCDC85C | coiled-coil domain containing 85C |
| 205175_s_at | Hs.567297 | KHK | ketohexokinase (fructokinase) |
| 213499_at | Hs.436847 | CLCN2 | chloride channel 2 |
| 219623_at | Hs.371585 | ACTR5 | ARP5 actin-related protein 5 homolog (yeast) |
| 218390_s_at | Hs.372309 | C10orf84 | chromosome 10 open reading frame 84 |
