# Supplementary material for: A Systematic Evaluation of Multi-Gene Predictors for the Pathological Response of Breast Cancer Patients to Chemotherapy
Source: PLoS One. 2012 Nov 21;7(11):e49529. doi: 10.1371/journal.pone.0049529 (PMC3504014; doi:10.1371/journal.pone.0049529)
Supplement: Table S12 — MGP-TFAC developed from the ER positive Hoeflich training set by the COXEN method. (DOC) [file pone.0049529.s012.doc]

Supplementary Table S12: MGP-TFAC developed from the ER positive Hoeflich training sets by the COXEN method.

| Probeset | UniGene.ID | Gene.Symbol | Gene.Title |
| --- | --- | --- | --- |
| 216981_x_at | Hs.632188 | SPN | sialophorin |
| 211160_x_at | Hs.509765 | ACTN1 | actinin, alpha 1 |
| 214627_at | Hs.279259 | EPX | eosinophil peroxidase |
| 206567_s_at | Hs.517044 | PHF20 | PHD finger protein 20 |
| 215442_s_at | Hs.160411 | TSHR | thyroid stimulating hormone receptor |
| 219564_at | Hs.463985 | KCNJ16 | potassium inwardly-rectifying channel, subfamily J, member 16 |
| 216787_at | Hs.677372 | NA | NA |
| 205782_at | Hs.567268 | FGF7 | fibroblast growth factor 7 |
| 204158_s_at | Hs.495985 | TCIRG1 | T-cell, immune regulator 1, ATPase, H+ transporting, lysosomal V0 subunit A3 |
| 204365_s_at | Hs.368884 | REEP1 | receptor accessory protein 1 |
| 210599_at | Hs.292336 | ZNF614 | zinc finger protein 614 |
