# Supplementary material for: A Systematic Evaluation of Multi-Gene Predictors for the Pathological Response of Breast Cancer Patients to Chemotherapy
Source: PLoS One. 2012 Nov 21;7(11):e49529. doi: 10.1371/journal.pone.0049529 (PMC3504014; doi:10.1371/journal.pone.0049529)
Supplement: Table S13 — MGP-TFAC developed from the ER negative Hoeflich training set by the COXEN method. (DOC) [file pone.0049529.s013.doc]

Supplementary Table S13: MGP-TFAC developed from the ER negative Hoeflich training sets by the COXEN method.

| Probeset | UniGene.ID | Gene.Symbol | Gene.Title |
| --- | --- | --- | --- |
| 208679_s_at | Hs.529303 | ARPC2 | actin related protein 2/3 complex, subunit 2, 34kDa |
| 218674_at | Hs.591760 | C5orf44 | chromosome 5 open reading frame 44 |
| 212320_at | Hs.636480 | TUBB | tubulin, beta |
| 213093_at | Hs.531704 | PRKCA | protein kinase C, alpha |
| 213696_s_at | Hs.301756 | MED8 | mediator complex subunit 8 |
| 210466_s_at | Hs.530412 | SERBP1 | SERPINE1 mRNA binding protein 1 |
| 212512_s_at | Hs.323213 | CARM1 | coactivator-associated arginine methyltransferase 1 |
| 208752_x_at | Hs.524599 | NAP1L1 | nucleosome assembly protein 1-like 1 |
| 212046_x_at | Hs.861 | MAPK3 | mitogen-activated protein kinase 3 |
| 213494_s_at | Hs.388927 | YY1 | YY1 transcription factor |
| 209975_at | Hs.12907 | CYP2E1 | cytochrome P450, family 2, subfamily E, polypeptide 1 |
| 203462_x_at | Hs.371001 | EIF3B | eukaryotic translation initiation factor 3, subunit B |
| 217294_s_at | Hs.517145 | ENO1 | enolase 1, (alpha) |
| 215381_at | Hs.338207 | MTOR | mechanistic target of rapamycin (serine/threonine kinase) |
| 213864_s_at | Hs.524599 | NAP1L1 | nucleosome assembly protein 1-like 1 |
| 204371_s_at | Hs.726172 | KHSRP | KH-type splicing regulatory protein |
| 211300_s_at | Hs.654481 | TP53 | tumor protein p53 |
| 219411_at | Hs.377416 | ELMO3 | engulfment and cell motility 3 |
| 214850_at | Hs.654588 | LOC100170939 | glucuronidase, beta pseudogene |
