# Supplementary material for: A Systematic Evaluation of Multi-Gene Predictors for the Pathological Response of Breast Cancer Patients to Chemotherapy
Source: PLoS One. 2012 Nov 21;7(11):e49529. doi: 10.1371/journal.pone.0049529 (PMC3504014; doi:10.1371/journal.pone.0049529)
Supplement: Table S14 — MGP-FEC developed from the ER positive Neve training set by the COXEN method. (DOC) [file pone.0049529.s014.doc]

Supplementary Table S14: MGP-FEC developed from the ER positive Neve training sets by the COXEN method.

| Probeset | UniGene.ID | Gene.Symbol | Gene.Title |
| --- | --- | --- | --- |
| 204291_at | Hs.600823 | ZNF518A | zinc finger protein 518A |
| 204798_at | Hs.606320 | MYB | v-myb myeloblastosis viral oncogene homolog (avian) |
| 208003_s_at | Hs.371987 | NFAT5 | nuclear factor of activated T-cells 5, tonicity-responsive |
| 212231_at | Hs.728146 | FBXO21 | F-box protein 21 |
| 215923_s_at | Hs.516306 | PSD4 | pleckstrin and Sec7 domain containing 4 |
| 213025_at | Hs.460232 | THUMPD1 | THUMP domain containing 1 |
| 203102_s_at | Hs.93338 | MGAT2 | mannosyl (alpha-1,6-)-glycoprotein beta-1,2-N-acetylglucosaminyltransferase |
| 64474_g_at | Hs.643452 | DGCR8 | DiGeorge syndrome critical region gene 8 |
| 204569_at | Hs.417022 | ICK | intestinal cell (MAK-like) kinase |
| 219166_at | Hs.231761 | C14orf104 | chromosome 14 open reading frame 104 |
| 200008_s_at | Hs.299055 | GDI2 | GDP dissociation inhibitor 2 |
| 202162_s_at | Hs.26703 | CNOT8 | CCR4-NOT transcription complex, subunit 8 |
| 222040_at | Hs.656277 | HNRNPA1 | heterogeneous nuclear ribonucleoprotein A1 |
| 217006_x_at | Hs.83190 | FASN | fatty acid synthase |
| 203552_at | Hs.130491 | MAP4K5 | mitogen-activated protein kinase kinase kinase kinase 5 |
| 204452_s_at | Hs.94234 | FZD1 | frizzled homolog 1 (Drosophila) |
| 211666_x_at | Hs.119598 | RPL3 | ribosomal protein L3 |
| 213639_s_at | Hs.513316 | ZNF500 | zinc finger protein 500 |
| 214472_at | Hs.532144 | HIST1H3D | histone cluster 1, H3d |
| 201990_s_at | Hs.591156 | CREBL2 | cAMP responsive element binding protein-like 2 |
