# Supplementary material for: A Systematic Evaluation of Multi-Gene Predictors for the Pathological Response of Breast Cancer Patients to Chemotherapy
Source: PLoS One. 2012 Nov 21;7(11):e49529. doi: 10.1371/journal.pone.0049529 (PMC3504014; doi:10.1371/journal.pone.0049529)
Supplement: Table S15 — MGP-FEC developed from the ER negative Neve training set by the COXEN method. (DOC) [file pone.0049529.s015.doc]

Supplementary Table S15: MGP-FEC developed from the ER negative Neve training sets by the COXEN method.

| Probeset | UniGene.ID | Gene.Symbol | Gene.Title |
| --- | --- | --- | --- |
| 202674_s_at | Hs.207631 | LMO7 | LIM domain 7 |
| 206506_s_at | Hs.368325 | SUPT3H | suppressor of Ty 3 homolog (S. cerevisiae) |
| 212528_at | Hs.570455 | PPPDE2 | PPPDE peptidase domain containing 2 |
| 218619_s_at | Hs.522639 | SUV39H1 | suppressor of variegation 3-9 homolog 1 (Drosophila) |
| 206230_at | Hs.443727 | LHX1 | LIM homeobox 1 |
| 210154_at | Hs.233119 | ME2 | malic enzyme 2, NAD(+)-dependent, mitochondrial |
| 211476_at | Hs.381047 | MYOZ2 | myozenin 2 |
| 207254_at | Hs.436893 | SLC15A1 | solute carrier family 15 (oligopeptide transporter), member 1 |
| 210764_s_at | Hs.8867 | CYR61 | cysteine-rich, angiogenic inducer, 61 |
| 211197_s_at | Hs.14155 | ICOSLG | inducible T-cell co-stimulator ligand |
| 211228_s_at | Hs.16184 | RAD17 | RAD17 homolog (S. pombe) |
| 204453_at | Hs.445019 | ZNF84 | zinc finger protein 84 |
| 218126_at | Hs.511067 | FAM82A2 | family with sequence similarity 82, member A2 |
| 219018_s_at | Hs.709288 | CCDC85C | coiled-coil domain containing 85C |
| 205774_at | Hs.1321 | F12 | coagulation factor XII (Hageman factor) |
| 213242_x_at | Hs.533721 | KIAA0284 | KIAA0284 |
| 222312_s_at | NA | NA | NA |
| 212156_at | Hs.88025 | VPS39 | vacuolar protein sorting 39 homolog (S. cerevisiae) |
| 204137_at | Hs.498160 | GPR137B | G protein-coupled receptor 137B |
| 212260_at | Hs.565319 | GIGYF2 | GRB10 interacting GYF protein 2 |
| 209386_at | Hs.351316 | TM4SF1 | transmembrane 4 L six family member 1 |
| 218125_s_at | Hs.445512 | CCDC25 | coiled-coil domain containing 25 |
| 209304_x_at | Hs.110571 | GADD45B | growth arrest and DNA-damage-inducible, beta |
| 218685_s_at | Hs.632721 | SMUG1 | single-strand-selective monofunctional uracil-DNA glycosylase 1 |
| 209150_s_at | Hs.91586 | TM9SF1 | transmembrane 9 superfamily member 1 |
| 209387_s_at | Hs.351316 | TM4SF1 | transmembrane 4 L six family member 1 |
| 55872_at | Hs.729072 | ZNF512B | zinc finger protein 512B |
| 207679_at | Hs.42146 | PAX3 | paired box 3 |
| 206024_at | Hs.2899 | HPD | 4-hydroxyphenylpyruvate dioxygenase |
| 201289_at | Hs.8867 | CYR61 | cysteine-rich, angiogenic inducer, 61 |
| 218360_at | Hs.529044 | RAB22A | RAB22A, member RAS oncogene family |
| 205175_s_at | Hs.567297 | KHK | ketohexokinase (fructokinase) |
| 220236_at | Hs.461183 | PDPR | pyruvate dehydrogenase phosphatase regulatory subunit |
| 215034_s_at | Hs.351316 | TM4SF1 | transmembrane 4 L six family member 1 |
| 203460_s_at | Hs.3260 | PSEN1 | presenilin 1 |
| 218154_at | Hs.118983 | GSDMD | gasdermin D |
| 217547_x_at | Hs.264345 | ZNF675 | zinc finger protein 675 |
| 215222_x_at | Hs.472475 | MACF1 | microtubule-actin crosslinking factor 1 |
| 207428_x_at | NA | NA | NA |
| 209920_at | Hs.471119 | BMPR2 | bone morphogenetic protein receptor, type II (serine/threonine kinase) |
| 202985_s_at | Hs.5443 | BAG5 | BCL2-associated athanogene 5 |
| 209305_s_at | Hs.110571 | GADD45B | growth arrest and DNA-damage-inducible, beta |
| 207574_s_at | Hs.110571 | GADD45B | growth arrest and DNA-damage-inducible, beta |
| 209997_x_at | Hs.491148 | PCM1 | pericentriolar material 1 |
| 209944_at | Hs.270869 | ZNF410 | zinc finger protein 410 |
| 217449_at | NA | NA | NA |
| 215515_at | NA | NA | NA |
| 202680_at | Hs.77100 | GTF2E2 | general transcription factor IIE, polypeptide 2, beta 34kDa |
| 218785_s_at | Hs.389104 | RABL5 | RAB, member RAS oncogene family-like 5 |
| 201994_at | Hs.326387 | MORF4L2 | mortality factor 4 like 2 |
| 205688_at | Hs.513305 | TFAP4 | transcription factor AP-4 (activating enhancer binding protein 4) |
| 216424_at | Hs.631659 | CD4 | CD4 molecule |
| 213305_s_at | Hs.368264 | PPP2R5C | protein phosphatase 2, regulatory subunit B', gamma |
| 214544_s_at | Hs.728237 | SNAP23 | synaptosomal-associated protein, 23kDa |
| 205743_at | Hs.56045 | STAC | SH3 and cysteine rich domain |
| 209922_at | Hs.530940 | BRAP | BRCA1 associated protein |
| 210929_s_at | NA | NA | NA |
| 216716_at | Hs.654423 | ABO | ABO blood group (transferase A, alpha 1-3-N-acetylgalactosaminyltransferase; transferase B, alpha 1-3-galactosyltransferase) |
| 207285_x_at | Hs.655225 | CSHL1 | chorionic somatomammotropin hormone-like 1 |
| 200919_at | Hs.524271 | PHC2 | polyhomeotic homolog 2 (Drosophila) |
| 210153_s_at | Hs.233119 | ME2 | malic enzyme 2, NAD(+)-dependent, mitochondrial |
| 209101_at | Hs.410037 | CTGF | connective tissue growth factor |
| 216133_at | NA | NA | NA |
| 205726_at | Hs.226483 | DIAPH2 | diaphanous homolog 2 (Drosophila) |
| 209819_at | Hs.494567 | HABP4 | hyaluronan binding protein 4 |
| 219631_at | Hs.600630/Hs.715867 | LRP12 | low density lipoprotein receptor-related protein 12 |
| 211974_x_at | Hs.479396 | RBPJ | recombination signal binding protein for immunoglobulin kappa J region |
| 203836_s_at | Hs.186486 | MAP3K5 | mitogen-activated protein kinase kinase kinase 5 |
| 203050_at | Hs.440968 | TP53BP1 | tumor protein p53 binding protein 1 |
| 210820_x_at | Hs.157113 | COQ7 | coenzyme Q7 homolog, ubiquinone (yeast) |
| 217642_at | Hs.65238 | RNF40 | ring finger protein 40 |
| 206945_at | Hs.551506 | LCT | lactase |
| 206636_at | Hs.655941 | RASA2 | RAS p21 protein activator 2 |
| 34406_at | Hs.525626 | PACS2 | phosphofurin acidic cluster sorting protein 2 |
| 212700_x_at | NA | NA | NA |
| 214529_at | Hs.406687 | TSHB | thyroid stimulating hormone, beta |
| 204076_at | Hs.444389 | ENTPD4 | ectonucleoside triphosphate diphosphohydrolase 4 |
| 205025_at | Hs.502330 | ZBTB48 | zinc finger and BTB domain containing 48 |
| 213513_x_at | Hs.529303 | ARPC2 | actin related protein 2/3 complex, subunit 2, 34kDa |
| 218937_at | Hs.592078 | ZNF434 | zinc finger protein 434 |
| 221200_at | NA | NA | NA |
| 210950_s_at | Hs.593928 | FDFT1 | farnesyl-diphosphate farnesyltransferase 1 |
| 214158_s_at | NA | NA | NA |
| 207654_x_at | Hs.348418 | DR1 | down-regulator of transcription 1, TBP-binding (negative cofactor 2) |
| 212970_at | Hs.479602 | APBB2 | amyloid beta (A4) precursor protein-binding, family B, member 2 |
| 218121_at | Hs.284279 | HMOX2 | heme oxygenase (decycling) 2 |
| 210256_s_at | Hs.655131/Hs.707569 | PIP5K1A | phosphatidylinositol-4-phosphate 5-kinase, type I, alpha |
| 204209_at | Hs.135997 | PCYT1A | phosphate cytidylyltransferase 1, choline, alpha |
| 214369_s_at | Hs.99491 | RASGRP2 | RAS guanyl releasing protein 2 (calcium and DAG-regulated) |
| 212204_at | Hs.511138 | TMEM87A | transmembrane protein 87A |
| 219450_at | Hs.107527 | C4orf19 | chromosome 4 open reading frame 19 |
| 215247_at | Hs.535619/Hs.721040 | LOC440895 | two pore channel 3 pseudogene |
| 210147_at | Hs.24976 | ART3 | ADP-ribosyltransferase 3 |
| 203944_x_at | Hs.159028 | BTN2A1 | butyrophilin, subfamily 2, member A1 |
| 213080_x_at | NA | NA | NA |
| 200937_s_at | Hs.532359 | RPL5 | ribosomal protein L5 |
| 202130_at | Hs.445511 | RIOK3 | RIO kinase 3 (yeast) |
| 206538_at | Hs.527021 | MRAS | muscle RAS oncogene homolog |
| 200742_s_at | Hs.523454 | TPP1 | tripeptidyl peptidase I |
| 215811_at | NA | NA | NA |
| 210747_at | Hs.409934/Hs.534322 | HLA-DQB1 | major histocompatibility complex, class II, DQ beta 1 |
| 211029_x_at | Hs.87191 | FGF18 | fibroblast growth factor 18 |
| 200625_s_at | Hs.370581/Hs.713078 | CAP1 | CAP, adenylate cyclase-associated protein 1 (yeast) |
| 202594_at | Hs.146585 | LEPROTL1 | leptin receptor overlapping transcript-like 1 |
| 217787_s_at | Hs.654649 | GALNT2 | UDP-N-acetyl-alpha-D-galactosamine:polypeptide N-acetylgalactosaminyltransferase 2 (GalNAc-T2) |
| 213292_s_at | Hs.487648 | SNX13 | sorting nexin 13 |
| 212778_at | Hs.525626 | PACS2 | phosphofurin acidic cluster sorting protein 2 |
| 211373_s_at | Hs.25363 | PSEN2 | presenilin 2 (Alzheimer disease 4) |
| 209750_at | Hs.37288 | NR1D2 | nuclear receptor subfamily 1, group D, member 2 |
| 209452_s_at | Hs.728787 | VTI1B | vesicle transport through interaction with t-SNAREs homolog 1B (yeast) |
| 209198_s_at | Hs.32984 | SYT11 | synaptotagmin XI |
| 209397_at | Hs.233119 | ME2 | malic enzyme 2, NAD(+)-dependent, mitochondrial |
| 205963_s_at | Hs.688238 | DNAJA3 | DnaJ (Hsp40) homolog, subfamily A, member 3 |
| 205128_x_at | Hs.201978 | PTGS1 | prostaglandin-endoperoxide synthase 1 (prostaglandin G/H synthase and cyclooxygenase) |
| 213798_s_at | Hs.370581/Hs.713078 | CAP1 | CAP, adenylate cyclase-associated protein 1 (yeast) |
| 214739_at | Hs.518414/Hs.659335 | LRCH3 | leucine-rich repeats and calponin homology (CH) domain containing 3 |
| 209707_at | Hs.178305 | PIGK | phosphatidylinositol glycan anchor biosynthesis, class K |
| 204488_at | Hs.531563 | DOLK | dolichol kinase |
| 204341_at | Hs.123534 | TRIM16 | tripartite motif containing 16 |
| 204603_at | Hs.498248 | EXO1 | exonuclease 1 |
| 208493_at | Hs.249171 | HOXA11 | homeobox A11 |
| 201912_s_at | Hs.528780 | GSPT1 | G1 to S phase transition 1 |
| 210101_x_at | Hs.136309 | SH3GLB1 | SH3-domain GRB2-like endophilin B1 |
| 210104_at | Hs.497353 | MED6 | mediator complex subunit 6 |
| 203540_at | Hs.514227 | GFAP | glial fibrillary acidic protein |
| 206439_at | Hs.435680 | EPYC | epiphycan |
| 218494_s_at | Hs.435126 | SLC2A4RG | SLC2A4 regulator |
| 219777_at | Hs.647105 | GIMAP6 | GTPase, IMAP family member 6 |
| 32723_at | Hs.172865 | CSTF1 | cleavage stimulation factor, 3' pre-RNA, subunit 1, 50kDa |
| 213175_s_at | Hs.83753 | SNRPB | small nuclear ribonucleoprotein polypeptides B and B1 |
| 206352_s_at | Hs.729134 | PEX10 | peroxisomal biogenesis factor 10 |
| 216393_at | Hs.14555/Hs.427927 | C10orf12 | chromosome 10 open reading frame 12 |
| 214083_at | Hs.368264 | PPP2R5C | protein phosphatase 2, regulatory subunit B', gamma |
| 202775_s_at | Hs.308171 | SFSWAP | splicing factor, suppressor of white-apricot homolog (Drosophila) |
| 204366_s_at | Hs.75782 | GTF3C2 | general transcription factor IIIC, polypeptide 2, beta 110kDa |
| 201361_at | Hs.13662 | TMEM109 | transmembrane protein 109 |
| 216612_x_at | NA | NA | NA |
| 209675_s_at | Hs.155218/Hs.718642 | HNRNPUL1 | heterogeneous nuclear ribonucleoprotein U-like 1 |
| 208725_at | Hs.429180 | EIF2S2 | eukaryotic translation initiation factor 2, subunit 2 beta, 38kDa |
| 216674_at | Hs.118727 | HES2 | hairy and enhancer of split 2 (Drosophila) |
| 220172_at | Hs.659439 | DCAF17 | DDB1 and CUL4 associated factor 17 |
| 213065_at | Hs.245798/Hs.527874 | ZFC3H1 | zinc finger, C3H1-type containing |
| 208709_s_at | Hs.584782 | NRD1 | nardilysin (N-arginine dibasic convertase) |
