# Supplementary material for: A Systematic Evaluation of Multi-Gene Predictors for the Pathological Response of Breast Cancer Patients to Chemotherapy
Source: PLoS One. 2012 Nov 21;7(11):e49529. doi: 10.1371/journal.pone.0049529 (PMC3504014; doi:10.1371/journal.pone.0049529)
Supplement: Table S16 — MGP-FEC developed from the ER positive Hoeflich training set by the COXEN method. (DOC) [file pone.0049529.s016.doc]

Supplementary Table S16: MGP-FEC developed from the ER positive Hoeflich training sets by the COXEN method.

| Probeset | UniGene.ID | Gene.Symbol | Gene.Title |
| --- | --- | --- | --- |
| 211933_s_at | Hs.632956 | HNRNPA3P1 | heterogeneous nuclear ribonucleoprotein A3 pseudogene 1 |
| 206809_s_at | Hs.632956 | HNRNPA3 | heterogeneous nuclear ribonucleoprotein A3 |
| 219800_s_at | Hs.645274 | THNSL1 | threonine synthase-like 1 (S. cerevisiae) |
| 214856_at | Hs.503178/Hs.705692 | SPTBN1 | spectrin, beta, non-erythrocytic 1 |
| 201299_s_at | Hs.728829 | MOBKL1B | MOB1, Mps One Binder kinase activator-like 1B (yeast) |
| 215241_at | Hs.91791 | ANO3 | anoctamin 3 |
| 210293_s_at | Hs.369373 | SEC23B | Sec23 homolog B (S. cerevisiae) |
| 214611_at | Hs.664641 | GRIK1 | glutamate receptor, ionotropic, kainate 1 |
| 201345_s_at | Hs.108332 | UBE2D2 | ubiquitin-conjugating enzyme E2D 2 (UBC4/5 homolog, yeast) |
| 212592_at | Hs.643431/Hs.715953 | IGJ | immunoglobulin J polypeptide, linker protein for immunoglobulin alpha and mu polypeptides |
| 221961_at | Hs.459649 | CLCN7 | chloride channel 7 |
| 218524_at | Hs.513268/Hs.615283 | E4F1 | E4F transcription factor 1 |
| 213264_at | Hs.546271 | PCBP2 | poly(rC) binding protein 2 |
| 211932_at | Hs.516539 | HNRNPA3 | heterogeneous nuclear ribonucleoprotein A3 |
| 216514_at | NA | NA | NA |
| 209896_s_at | Hs.506852 | PTPN11 | protein tyrosine phosphatase, non-receptor type 11 |
| 203261_at | Hs.158427/Hs.721134 | DCTN6 | dynactin 6 |
