# Supplementary material for: A Systematic Evaluation of Multi-Gene Predictors for the Pathological Response of Breast Cancer Patients to Chemotherapy
Source: PLoS One. 2012 Nov 21;7(11):e49529. doi: 10.1371/journal.pone.0049529 (PMC3504014; doi:10.1371/journal.pone.0049529)
Supplement: Table S17 — MGP-FEC developed from the ER negative Hoeflich training set by the COXEN method. (DOC) [file pone.0049529.s017.doc]

Supplementary Table S17: MGP-FEC developed from the ER negative Hoeflich training sets by the COXEN method.

| Probeset | UniGene.ID | Gene.Symbol | Gene.Title |
| --- | --- | --- | --- |
| 211031_s_at | Hs.647018 | CLIP2 | CAP-GLY domain containing linker protein 2 |
| 213564_x_at | Hs.446149 | LDHB | lactate dehydrogenase B |
| 206972_s_at | Hs.271809 | GPR161 | G protein-coupled receptor 161 |
| 201030_x_at | Hs.446149 | LDHB | lactate dehydrogenase B |
| 219266_at | Hs.407694 | ZNF350 | zinc finger protein 350 |
| 206648_at | Hs.590944 | ZNF571 | zinc finger protein 571 |
| 211958_at | Hs.607212 | IGFBP5 | insulin-like growth factor binding protein 5 |
| 214206_at | Hs.32234 | NA | NA |
| 209878_s_at | Hs.502875 | RELA | v-rel reticuloendotheliosis viral oncogene homolog A (avian) |
| 203424_s_at | Hs.607212 | IGFBP5 | insulin-like growth factor binding protein 5 |
| 209910_at | Hs.180408 | SLC25A16 | solute carrier family 25 (mitochondrial carrier; Graves disease autoantigen), member 16 |
| 201853_s_at | Hs.153752 | CDC25B | cell division cycle 25 homolog B (S. pombe) |
| 201323_at | Hs.346868 | EBNA1BP2 | EBNA1 binding protein 2 |
| 209782_s_at | Hs.528006 | DBP | D site of albumin promoter (albumin D-box) binding protein |
| 209975_at | Hs.12907 | CYP2E1 | cytochrome P450, family 2, subfamily E, polypeptide 1 |
| 210457_x_at | Hs.518805 | HMGA1 | high mobility group AT-hook 1 |
| 205479_s_at | Hs.77274 | PLAU | plasminogen activator, urokinase |
| 34210_at | Hs.276770 | CD52 | CD52 molecule |
| 203425_s_at | Hs.607212 | IGFBP5 | insulin-like growth factor binding protein 5 |
| 212512_s_at | Hs.323213 | CARM1 | coactivator-associated arginine methyltransferase 1 |
| 209934_s_at | Hs.584884 | ATP2C1 | ATPase, Ca++ transporting, type 2C, member 1 |
| 203411_s_at | Hs.594444 | LMNA | lamin A/C |
| 218674_at | Hs.591760 | C5orf44 | chromosome 5 open reading frame 44 |
| 215961_at | Hs.1321 | NA | NA |
| 218812_s_at | Hs.363308 | ORAI2 | ORAI calcium release-activated calcium modulator 2 |
| 202708_s_at | Hs.2178 | HIST2H2BE | histone cluster 2, H2be |
